# Effects of High-Intensity Interval Training on Cardiorespiratory Fitness and Cardiometabolic Health in Real-World Settings: A Systematic Review and Meta-Analysis

**DOI:** 10.3390/sports14070311

**Published:** 2026-07-22

**Authors:** Julijan Stefanovic, Valentina Victoria Werndle, Dejan Reljic

**Affiliations:** 1 Medical Faculty, Friedrich-Alexander-University Erlangen-Nürnberg, 91054 Erlangen, Germany; julijan.stefanovic@fau.de (J.S.); valentina.werndle@fau.de (V.V.W.); 2 Department of Medicine 1–Gastroenterology, Pneumology and Endocrinology, University Hospital Erlangen, Friedrich-Alexander University Erlangen-Nürnberg, 91054 Erlangen, Germany; 3 Hector-Center for Nutrition, Exercise and Sports, Department of Medicine 1, University Hospital Erlangen, Friedrich-Alexander University Erlangen-Nürnberg, 91054 Erlangen, Germany

**Keywords:** HIIT, interval training, real-world interventions, VO_2max_, metabolic health, exercise effectiveness

## Abstract

High-intensity interval training (HIIT) is an effective exercise strategy for improving cardiorespiratory fitness (CRF) and cardiometabolic health in laboratory settings, yet its effectiveness in real-world conditions remains less clear. This systematic review and meta-analysis examined the effects of real-world HIIT on CRF and cardiometabolic outcomes in adults. We searched five major databases (inception to December 2025) for controlled and non-controlled reports of ≥1 cardiorespiratory or cardiometabolic outcome. Random-effects models were used to calculate weighted mean differences (WMD), with subgroup and meta-regression analyses to explore moderators. Thirty-one studies (n = 1119) were included. In the pooled pre–post analyses, HIIT was associated with significant within-group improvements in VO_2max/peak_ (3.9 mL/kg/min), systolic (−4 mmHg) and diastolic blood pressure (−3 mmHg), triglycerides (−8 mg/dL), total cholesterol (−9 mg/dL), LDL (−10 mg/dL), and waist circumference (−1.7 cm) across interventions. In controlled comparisons, HIIT was superior to passive controls for VO_2max/peak_ (6.9 mL/kg/min) and waist circumference (−2.9 cm), while superiority over active controls was limited to VO_2max/peak_ (1.1 mL/kg/min). Moderator analyses revealed larger cardiometabolic benefits in overweight and clinical populations, and greater improvements in fasting glucose and blood pressure with higher exercise volume and longer intervention duration. In conclusion, real-world HIIT can effectively improve CRF and selected cardiometabolic outcomes across diverse adult populations. Specific cardiometabolic benefits appear to be pronounced in overweight, clinical, and previously untrained individuals. Notably, substantial heterogeneity and moderate methodological quality warrant cautious interpretation.

## 1. Introduction

Physical exercise is widely recognized as a cornerstone of health promotion and disease prevention. Extensive epidemiological and experimental evidence demonstrates that regular exercise can improve physical and mental health, enhance quality of life, and reduce the risk of premature mortality [1]. These benefits are reflected in numerous national and international public health recommendations, which advise adults to accumulate at least 150 min of moderate-intensity aerobic activity or 75 min of vigorous-intensity aerobic activity per week, supplemented by muscle-strengthening activities on at least two days per week to support overall health and functional capacity [2].

Despite these well-established guidelines, physical inactivity remains a major global health challenge. Recent surveillance data indicate that approximately one third (31%) of the world’s adult population does not meet the recommended physical activity levels, representing an increase of nearly eight percentage points since the early 2000s and suggesting that the global prevalence of insufficient physical activity continues to rise [3]. These trends are worrisome, as physical inactivity is associated with an increased risk of morbidity and mortality from cardiovascular and several other chronic diseases [4]. Thus, identifying exercise strategies that are both effective and feasible to implement in everyday life represents a high priority for public health and preventive medicine.

In this context, high-intensity interval training (HIIT) has emerged as a potent and time-efficient exercise modality. HIIT is typically characterized by repeated bouts of vigorous to near-maximal exercise interspersed with periods of active recovery or rest. Depending on protocol design, work intervals may range from a few seconds to several minutes [5]. Extremely brief supramaximal variants, commonly referred to as sprint interval training (SIT), represent a related form of HIIT that involves short “all-out” efforts separated by respective recovery periods [6]. While historically implemented in competitive sports settings to augment endurance performance, a growing body of evidence demonstrates that HIIT can elicit significant improvements in cardiorespiratory fitness (CRF) [7] and multiple cardiometabolic risk markers [8,9,10,11] across diverse populations. Notably, it has been consistently reported that the beneficial health effects of HIIT seem to be comparable or even superior to those achieved through traditional moderate-intensity continuous training (MICT), despite requiring substantially less total exercise time [7,9,10,11].

However, most evidence supporting the health benefits of HIIT originates from controlled laboratory or clinical studies conducted within specific research facilities. While such experimental designs are essential for elucidating physiological mechanisms, they may not necessarily reflect how HIIT is implemented in everyday environments such as community fitness programs, sports clubs, workplaces, or unsupervised home-based exercise. This distinction highlights the broader methodological difference between exercise efficacy and exercise effectiveness. Specifically, efficacy refers to the extent to which an intervention produces the intended effect under ideal, tightly controlled experimental conditions, whereas effectiveness reflects whether comparable benefits are achieved when the intervention is implemented under routine real-world circumstances [12]. In exercise science, this distinction is particularly relevant because factors such as time constraints, fluctuating motivation, variable supervision, and heterogeneous participant characteristics may influence both adherence and physiological adaptations. In laboratory-based studies, compliance rates are typically reported to be higher due to more intensive supervision and strict protocol control. In contrast, real-world exercise programs frequently involve greater variability in training intensity, adherence, and program delivery [13,14].

Translating HIIT from tightly controlled laboratory studies into routine practice is therefore an important but insufficiently explored area of research. Importantly, “real-world” implementation should not be interpreted solely as exercise performed outside a laboratory. Rather, effectiveness reflects the extent to which interventions are delivered under conditions that resemble routine practice, including variability in supervision, participant autonomy, adherence, intervention delivery, and environmental context. Consequently, real-world HIIT encompasses a spectrum of implementation settings, each differing in their degree of ecological validity. Understanding whether HIIT remains effective across these diverse contexts is essential for evaluating its translational potential as a scalable public health strategy. Therefore, the present systematic review and meta-analysis evaluated the effects of HIIT performed under real-world implementation conditions on CRF and key cardiometabolic health outcomes in adults.

## 2. Materials and Methods

### 2.1. Search and Screening Procedures

**Figure 1:**
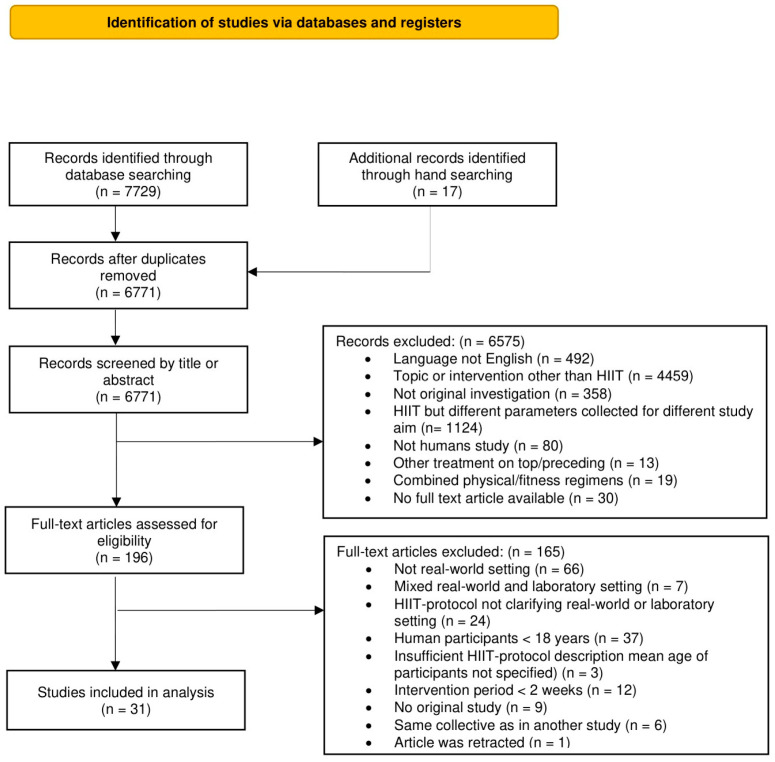
Flow diagram of the systematic literature search.

This meta-analysis was conducted in accordance with the Preferred Reporting Items for Systematic Reviews and Meta-Analyses (PRISMA) 2020 guidelines [15,16] and was prospectively registered in the International Prospective Register of Systematic Reviews (PROSPERO; registration number: CRD420250639241). The literature search and study selection procedures were conducted independently by two reviewers (J.S. and V.V.W.). Electronic database searches were performed in PubMed/MEDLINE, Web of Science, Scopus, SPORTDiscus, and the Cochrane Central Register of Controlled Trials (CENTRAL) from database inception until December 2025. Search terms related to HIIT and CRF and cardiometabolic health outcomes were combined using predefined Boolean operators and applied across multiple search levels including intervention terms, health outcomes, and real-world implementation contexts (Table S1). In addition, reference lists of relevant systematic reviews and included studies were manually screened to identify additional eligible publications. All duplicates were removed prior to screening. The remaining studies were screened sequentially based on (i) titles, (ii) abstracts, and (iii) full-text articles to determine eligibility. Disagreements between reviewers during the screening process were resolved through discussion and consensus, and when necessary, consultation with a third independent reviewer (D.R.). The overall study selection process is presented in Figure 1.

### 2.2. Inclusion and Exclusion Criteria

Eligibility criteria were defined according to the PICOS framework [17]. The Population (P) included adult participants (≥18 years) irrespective of sex, health status, or body mass index (BMI). Studies involving clinical populations as well as apparently healthy individuals were both considered eligible. Studies involving children, adolescents, or elite athletes were excluded. Trained individuals and recreational athletes were considered eligible, provided that the intervention was conducted under routine practice or training conditions consistent with the real-world inclusion criteria. To account for the different baseline fitness levels, adaptive capacity, and cardiometabolic risk profiles across participant types, training status was prespecified as a moderator variable and analyzed through subgroup analyses stratifying participants into healthy trained, healthy untrained, and clinical or at-risk categories.

The Intervention (I) involved HIIT interventions conducted in real-world settings. HIIT was defined according to contemporary exercise science recommendations as repeated bouts of vigorous- to near-maximal intensity exercise interspersed with periods of active or passive recovery [5]. Study inclusion was not based solely on the terminology used by the original authors. Rather, eligible interventions were required to exhibit the defining structural characteristics of HIIT, namely repeated high-intensity work intervals separated by planned recovery periods. Accordingly, all established HIIT variants including sprint interval training (SIT), low-volume HIIT, functional HIIT, repeated-sprint training, and Tabata protocols were considered eligible, provided they were explicitly designed to elicit vigorous or near-maximal physiological stress and implemented in real-world environments. For readability reasons, all these subtypes are subsequently referred to as “HIIT”, unless otherwise noted. Studies describing interval exercise that did not include a clearly intended high-intensity stimulus or represented moderate-intensity interval exercise were excluded. Real-world HIIT interventions were defined as exercise programs implemented under routine practice conditions rather than tightly controlled laboratory efficacy settings. Eligible interventions were conducted in applied environments such as workplaces, community fitness facilities, sports clubs, rehabilitation services, home-based settings, outdoor environments, or other non-laboratory contexts intended to reflect usual exercise practice. Importantly, real-world status was not determined solely by physical location but also by the pragmatic nature of intervention delivery, including varying degrees of supervision, participant autonomy, implementation flexibility, and ecological validity. Accordingly, both supervised and unsupervised programs were eligible provided that the intervention was implemented within a routine practice environment rather than a dedicated exercise physiology laboratory.

The Comparator (C) included one of the following comparator conditions: non-exercise control groups, usual care or habitual activity, or alternative exercise interventions (e.g., MICT, resistance training, etc.). Studies without a comparator group were also included if pre–post intervention data were available.

The primary Outcomes (O) included maximal or peak oxygen uptake (VO_2max_ or VO_2peak_), systolic blood pressure (SBP), diastolic blood pressure (DBP), fasting glucose (FG), total cholesterol (TC), high-density lipoprotein cholesterol (HDL), low-density lipoprotein cholesterol (LDL), triglycerides, fasting insulin, and Homeostasis Model Assessment of Insulin Resistance (HOMA-IR). Secondary outcomes included body composition indices, including body weight, BMI, body fat percentage (BF%), and waist circumference (WC). When studies did not report outcomes of interest with sufficient detail, the corresponding authors were contacted to request the required data. If the information remained unavailable after the initial request and a follow-up reminder within 2–4 weeks, the study was excluded from the quantitative synthesis.

The Study Design (S) included randomized controlled trials (RCTs), non-randomized controlled trials, and controlled intervention studies with a comparator group. In addition, single-group pre–post intervention studies without a control condition were eligible for inclusion in pooled pre–post analyses, provided they reported pre- and post-intervention outcome data for at least one primary or secondary outcome of interest. This decision was made to maximize the use of available real-world effectiveness data, given the recognized scarcity of controlled trials in applied implementation settings. It is acknowledged that single-group designs and controlled trials address distinct inferential questions: Pre–post analyses estimate the magnitude of within-group change under real-world conditions, whereas controlled comparisons provide counterfactual estimates of HIIT effectiveness relative to non-exercise or alternative exercise conditions. These analytic streams are therefore reported and interpreted separately throughout. Observational studies, case reports, reviews, conference abstracts without full text, and non-English publications were excluded.

### 2.3. Data Extraction

Data extraction was performed independently by two reviewers (J.S., V.V.W.) using a standardized extraction template. The following information was extracted from each study: study characteristics (author, year), participant characteristics (sample size, age, sex, training/health status), HIIT protocol characteristics (exercise intensity, session duration, training frequency, intervention duration) supervision status (supervised, partially supervised, or unsupervised), training setting (e.g., workplace, community, home, outdoor, fitness center) and other implementation characteristics relevant to real-world delivery, comparator intervention (if applicable), and pre- and post-intervention outcome values (means and standard deviations). Total session duration was extracted as reported by the authors and included warm-up, recovery phases between interval bouts and cool-down periods. For VO_2max_/VO_2peak_, the method of assessment was additionally extracted and categorized as either direct measurement via incremental cardiopulmonary exercise testing with respiratory gas analysis or field-based estimation (e.g., multistage shuttle run). Any discrepancies between reviewers were resolved through discussion and consensus.

### 2.4. Assessment of Methodological Quality and Sensitivity Analyses

The methodological quality and risk of bias of included studies were independently assessed by two reviewers (J.S., V.V.W.) via design-specific tools. RCTs were evaluated using the Physiotherapy Evidence Database (PEDro) scale, which assesses key methodological study domains [18]. Accordingly, studies were categorized as: excellent (9–10 points), good (6–8 points), fair (4–5 points), or poor (<4 points) quality. Single-group pre–post studies without a control group were assessed using the NIH Quality Assessment Tool [19]. Here, a numerical rating is applied by summing the number of criteria fulfilled (maximum = 12), with studies categorized as poor (0–4), fair (5–8), or good quality (9–12). This approach was chosen to ensure appropriate appraisal criteria for each study design and to avoid bias associated with applying a single tool across heterogeneous methodologies. Disagreements were resolved through consensus, and when necessary, by consultation with a third reviewer (D.R.). Additionally, sensitivity analyses were undertaken for all outcomes using a leave-one-out approach, in which the meta-analysis was repeated with each study sequentially removed in order to identify potential influence of individual studies.

### 2.5. Statistical Analyses

Given the heterogeneous study designs represented in the included evidence base, three complementary meta-analytic approaches were applied, each addressing a distinct inferential question. First, pooled pre–post analyses were conducted across all included studies to estimate the overall within-group magnitude of change associated with real-world HIIT. These analyses capture the observed effect size under real-world conditions but do not control for non-specific effects. Thus, they should be interpreted as descriptive estimates of real-world responsiveness rather than controlled effectiveness evidence. Second, separate comparative analyses were performed for studies that included a passive control group (non-exercise or usual care), providing a direct controlled estimate of HIIT effectiveness relative to non-exercise behavior. Third, comparative analyses were performed for studies that included an active control condition (e.g., MICT), enabling assessment of the relative advantage of HIIT over other exercise modalities. It is important to note that these three analytic streams are not inferentially equivalent. Pre–post estimates reflect uncontrolled within-group change, while comparative analyses provide the controlled evidence base from which causal conclusions about HIIT effectiveness can be drawn.

For pre–post analyses, effect sizes were calculated as within-group weighted mean differences (WMDs) between post- and pre-intervention values with corresponding 95% confidence intervals (CIs) using random-effects models in order to account for expected clinical and methodological heterogeneity across studies. Where studies reported the standard deviation of change scores (SD_change_) directly, these were used. Where SD_change_ was not reported, it was estimated from the formula:

SD_change_ = √(SDpre^2^ + SDpost^2^ − 2 × r × SDpre × SDpost),

applying a conservative default correlation of r = 0.7 consistent with established meta-analytic recommendations [20]. For comparative analyses, WMDs were calculated as the difference between the HIIT and control group change scores. In pooled pre–post analyses, multiple arms from the same study were entered as independent effect sizes, with the understanding that this approach may slightly underestimate true between-study variance. In comparative analyses involving a shared control group, the control group sample size was divided equally across the relevant HIIT arms to avoid artificially inflating precision due to unit-of-analysis error [20].

Statistical heterogeneity among studies was assessed using Cochran’s Q test and quantified using the I^2^ statistic, with thresholds interpreted according to Cochrane recommendations [20]. Specifically, values of 25%, 50%, and 75% were considered to represent low, moderate, and high heterogeneity, respectively. Prespecified subgroup analyses were conducted to examine potential sources of heterogeneity related to intervention and population characteristics, including participant type (healthy trained, healthy untrained, and clinical populations), HIIT modality (walking/running, cycling, stair climbing, sports specific exercise, functional training, and mixed exercises), exercise intensity (<90% HR_max_, 90–99% HR_max_, and 100% HR_max_/all-out), and study setting (outdoor, gym-based, and workplace/home-based). In accordance with recommendations [20], subgroup analyses were conducted only when ≥3 studies were available per subgroup category.

Additionally, random-effects meta-regression analyses were conducted to further explore potential moderators of intervention effects. Continuous moderator variables included baseline age, intervention duration, weekly training frequency, and exercise volume (min/week) where available. Each moderator was examined separately in univariable models, with regression coefficients representing the change in effect size associated with a one-unit increase in the respective moderator variable. Meta-regressions were performed only when ≥10 independent effect sizes were available for a given outcome to ensure adequate statistical power and reliability [20].

Potential publication bias was assessed visually by inspection of funnel plots for asymmetry and statistically using Egger’s regression test [21]. Sensitivity analyses were additionally conducted by sequentially removing individual studies to examine the robustness of pooled estimates. All statistical analyses were performed using Comprehensive Meta-Analysis software (version 4.0; Biostat Inc., Englewood, NJ, USA). A two-sided *p*-value < 0.05 was considered to indicate statistical significance unless otherwise specified.

## 3. Results

### 3.1. Study Selection

The initial database search identified 7712 records (PubMed/MEDLINE: n = 968; Web of Science: n = 446; Scopus: n = 51; SPORTDiscus: n = 5441; Cochrane Central Register of Controlled Trials: n = 806). Additionally, 17 records were identified through manual searches of reference lists and related articles yielding a total number of 7729 records. After removal of duplicates, 6771 records remained and were screened based on title and abstract. During this screening stage, 6575 records were excluded as they did not match the predefined inclusion criteria. Subsequently, a total of 196 full-text articles were assessed for eligibility and of these, 31 studies met the inclusion criteria and were included in the qualitative and quantitative synthesis (Figure 1).

### 3.2. Methodological Quality of Included Studies

The overall methodological quality of the included studies was rated as moderate to good. Among the 25 RCTs, 18 were classified as high (PEDro scores 6–8), six as fair (scores 4–5), and one as low quality (score 3), yielding a mean PEDro score of 6.2 (Table S2). Among the six single-group pre–post studies without control group, five were rated as fair (scores 5–8) and one as good quality (score 9) based on the NIH quality assessment tool (Table S3).

### 3.3. Participant and Study Characteristics

Overall, data from 1119 participants were included in the analysis. Sample sizes ranged from 5 to 98 participants per study, with a mean of 19 participants per intervention arm. The majority of studies enrolled mixed-sex cohorts (n = 16), while five studies included only women and 10 studies included only men. The mean participant age was 34 years, ranging from 20 to 70 years. Baseline CRF and body weight status varied across studies, with a mean VO_2max/peak_ of 35.3 mL/kg/min (range: 19.8–53.3 mL/kg/min) and a mean BMI of 26.3 kg/m^2^ (range: 19.9–33.2 kg/m^2^). Study populations comprised a heterogeneous mix of participant groups, including healthy physically active or trained individuals (n = 8), healthy but untrained or sedentary individuals (n = 14), and clinical or at-risk populations (n = 8). The latter included participants with overweight or obesity (n = 6), Parkinson’s disease (n = 1), and amphetamine-type stimulant dependence (n = 1).

Across the included studies, seven studies implemented more than one HIIT protocol, resulting in a total of 39 HIIT intervention arms included in the analyses. Comparator conditions varied, with nine studies comparing HIIT to passive control groups and 14 studies comparing HIIT to active control interventions, such as walking or moderate-intensity ergometer training. The mean intervention duration was 11 weeks (range: 2–52 weeks). Exercise frequency ranged from one to three sessions per week, with three sessions per week being the most commonly prescribed frequency. Session duration varied considerably, ranging from 5 to 69 min, with a mean duration of 29.8 min per session. A wide range of exercise modalities was employed across studies, including walking-/running-based protocols (n = 12), cycling (n = 6), sports-specific training (e.g., front crawl swimming or shadowboxing; n = 4), functional exercise protocols (n = 4), mixed-modality interventions (n = 3), and stair climbing (n = 2). Prescribed interval intensities ranged from vigorous to maximal exercise, with the majority of studies prescribing intensities between 90–99% HR_max_ (n = 15) or maximal (“all-out”) effort (n = 16). Lower prescribed heart-rate targets (<90% HR_max_, n = 5) generally reflected established low-volume or functional HIIT protocols that nevertheless fulfilled the predefined eligibility criteria. Levels of supervision ranged from fully supervised (n = 13), over partially supervised (n = 1) to entirely unsupervised (n = 17) interventions, illustrating the considerable diversity of real-world implementation represented within the included evidence base. The included interventions represented a broad range of real-world implementation contexts, including workplace-based programs (n = 6), fitness centers (n = 5), home-based interventions (n = 2), outdoor programs (n = 7), specific sports facilities (n = 7), and community-based settings (n = 4).

Participant adherence was generally high, with a mean dropout rate of 14.9% across studies. Table 1 summarizes the included participant and study characteristics.

### 3.4. Meta-Analysis Results

#### 3.4.1. Pre–Post Effects of Real-World HIIT Interventions

**Figure 2:**
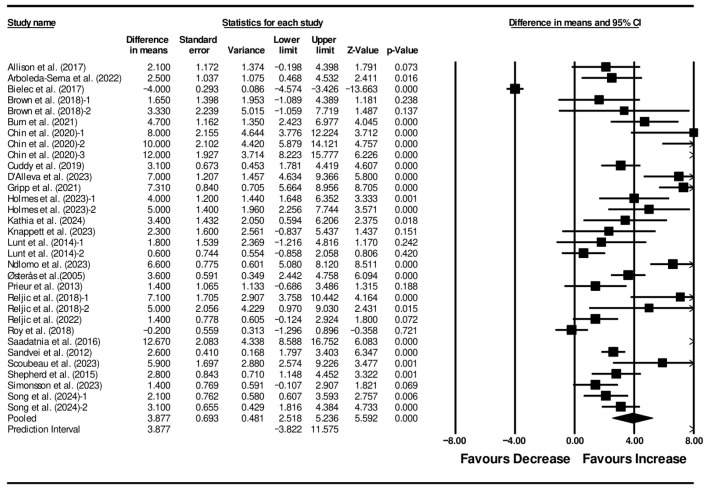
Forest plot of real-world HIIT effects on VO_2max/peak_. Weighted mean differences and 95% confidence intervals. Squares represent the individual study effect estimates, horizontal lines represent the 95% confidence intervals, and diamonds represent the pooled effect estimates. Arrows indicate confidence intervals extending beyond the displayed axis range [22,23,24,25,26,27,28,29,30,31,32,33,34,35,36,37,38,39,40,41,42,43,44,45,46].

**Figure 3:**
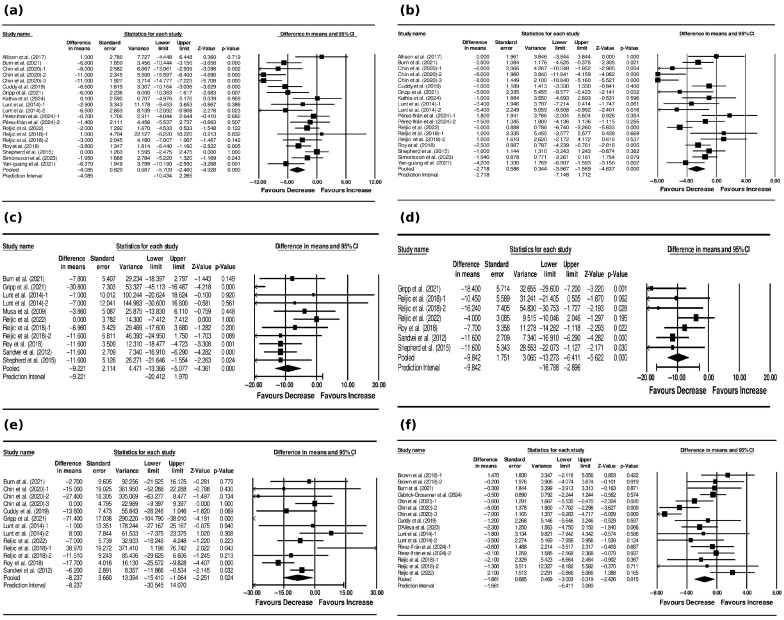
Forest plots of real-world HIIT effects on (**a**) systolic blood pressure, (**b**) diastolic blood pressure, (**c**) total cholesterol, (**d**) LDL cholesterol, (**e**) triglycerides, and (**f**) waist circumference. Weighted mean differences and 95% confidence intervals. Squares represent the individual study effect estimates, horizontal lines represent the 95% confidence intervals, and diamonds represent the pooled effect estimates. Arrows indicate confidence intervals extending beyond the displayed axis range [22,25,26,27,28,29,30,32,34,38,39,40,41,42,44,45,50,51,52].

Pooled analyses demonstrated a significant increase in VO_2max/peak_ by 3.9 mL/kg/min (95% CI: 2.5 to 5.2 mL/kg/min, *p* < 0.001; *I*^2^ = 95%; k = 32; Figure 2). Significant reductions were observed in SBP (−4 mmHg; 95% CI: −6 to −3 mmHg, *p* < 0.001; *I*^2^ = 69%; k = 19; Figure 3a) and DBP (−3 mmHg; 95% CI: −4 to −2 mmHg, *p* < 0.001; *I*^2^ = 68%; k = 19; Figure 3b).

Reductions were found for TC (−9 mg/dL; 95% CI: −13 to −5 mg/dL, *p* < 0.001; *I*^2^ = 45%; k = 11; Figure 3c), LDL (−10 mg/dL; 95% CI: −13 to −6 mg/dL, *p* < 0.001; *I*^2^ = 20%; k = 7; Figure 3d), and triglycerides (−8 mg/dL; 95% CI: −15 to −1 mg/dL, *p* = 0.024; *I*^2^ = 66%; k = 13; Figure 3e). No significant effects were detected for FG (0 mg/dL; 95% CI: −2 to 2 mg/dL, *p* = 0.977; *I*^2^ = 87%; k = 12), HDL (0 mg/dL; 95% CI: −0.1 to 0.2 mg/dL, *p* = 0.533; *I*^2^ = 52%; k = 15), insulin (−1 mg/dL; 95% CI: −2 to 0 mg/dL, *p* = 0.062; *I*^2^ = 66%; k = 7), and HOMA-IR (−0.1 units; 95% CI: −7.5 to 7.3 units, *p* = 0.978; *I*^2^ = 79%; k = 9). Body weight showed a borderline but non-significant reduction (−1.0 kg; 95% CI: −2.0 to 0 kg, *p* = 0.059; *I*^2^ = 36%; k = 21). However, significant reductions were found for BMI (−0.4 kg/m^2^; 95% CI: −0.8 to 0 kg/m^2^, *p* = 0.041; *I*^2^ = 64%; k = 23), BF% (−1.5%; 95% CI: −2.1 to −0.9%, *p* < 0.001; *I*^2^ = 56%; k = 24), and WC (−1.7 cm; 95% CI: −3.0 to −0.3 cm, *p* = 0.015; *I*^2^ = 65%; k = 16; Figure 3f).

**Table 1:** Overview of Participant and Intervention Characteristics.

Study	Participants	Groups	Sample Size	Age (Years)	Duration (Weeks)	Exercise Type	Setting/Supervision	Protocol Description
Allison et al. [22]	sedentary women	SIT	11	26 ± 11	6	stair climbing	stairwell/S	3 × 20 s all-out, divided by 2 min active recovery; total session duration: 10 min; 3 sessions/week
Arboleda-Serna et al. [23]	overweight women	HIIT MICT	16 19	29.7 ± 7.2 29.5 ± 8.1	8	walking, jogging, running	outdoor (sports field)/US	HIIT: 15 × 30 s @ 92.5% HR_max_, divided by 60 s active recovery; total session duration: not specified; 3 sessions/week MICT: a total of 35 min @ 70% HR_max_; 3 sessions/week
Bielec et al. [24]	experienced male swimmers	SIT	7	21.5 ± 0.5	2	swimming (front crawl)	indoor swimming pool/US	12 × 25 m all-out, rest periods not specified; total session duration: not specified; 3 sessions/week
Brown et al. [25]	recreationally active females	HIIT-1 HIIT-2	9 7	23.8 ± 6.4 22.0 ± 2.8	12	HIIT-1: total body exercises HIIT-2: rowing	university recreation center/US	Both HIIT protocols: 6× 60 s @ ≥90% HR_max_, divided 3 min rest; total session duration: ~60 min; 3 sessions/week
Burn et al. [26]	healthy male and female employees	HIIT CON	30 24	46 ± 9 46 ± 12	8	stair climbing, stepping, boxing	meeting room or outside the workplace/US	HIIT: 4–7× 60 s, divided by 75 s rest; total session duration: 15–22 min; 3 sessions/week CON: inactive
Cebrick-Grossman et al. [47]	sedentary, obese females	HIIT CON	5 7	46.1 ± 9.6 46.1 ± 9.6	12	HIIT: total body exercises CON: walking	workplace/US	HIIT: repeated high-intensity total body exercises guided by DVD-program; total session duration: 15 min; 3–5 sessions/week CON_)_: instructed to walk 5000–10,000 steps/day
Chin et al. [27]	overweight or obese men	HIIT-1 HIIT-2 HIIT-3 CON	14 15 31 27	22.8 ± 3.1 (mean)	8	shuttle runs	rooftop-covered field/S	HIIT-1: 1 session/week HIIT-2: 2–3 sessions/week HIIT-3: 3 sessions/week All HIIT protocols:12× 1 min @ 90% HR_max_, divided by 1 min active recovery; total session duration: not specified CON: no intervention
Cuddy et al. [28]	inactive men and women	HIIT MICT	16 16	40.8 ± 10.8 42.2 ± 9.7	8	cycle ergometer	workplace/S	HIIT: 2× 20 s sprints, divided by 3 min rest; total session duration: 10 min; 2–4 sessions/week MICT: a total of 30 min @ 50–65% HRR; 5 sessions/week
D’Alleva et al. [29]	obese males	HIIT	16	38.3 ± 7.1	12	walking/running	flat terrain track, city circuit/US	5–7× 2 min @ 95% VO_2peak_, divided by 1 min active recovery; total session duration: 45 min; 3 sessions/week
Eather et al. [48]	sedentary male and female employees	HIIT CON	25 23	43.0 ± 10.7 (mean)	8	combined aerobic/strength exercises	workplace/S	HIIT: 8× 30–40 s @ >85% HR_max_, divided by 30–40 s rest; total session duration: 8 min; 2–3 sessions/week CON: continuation of usual lifestyle
Gripp et al. [30]	healthy males and females	HIIT MICT	14 13	38 ± 6 39 ± 5	8	running	outdoor/S	HIIT: 7–10× 200 m runs @ 92.5% V_max_, divided by 1 min passive recovery; total session duration: 18 min; 3 sessions/week MICT: 3500–5000 m running @ 67.5% V_max_; total session duration: 31 min; 3 sessions/week
Holmes et al. [31]	recreationally active females	HIIT-1 HIIT-2	15 15	20.7 ± 2.4 22.7 ± 3.5	8	HIIT-1: plyometric exercise HIIT-2: stationary cycling	university recreational center/S	HIIT-1: 4× 1–2 min all-out, divided by 30 s rest or active recovery; total session duration: 30 min; 2 sessions/week HIIT-1: 20–80 s all-out, divided by 10–60 s rest or active recovery; total session duration: 30 min; 2 sessions/week
Kathia et al. [32]	males and females with Parkinson’s disease	HIIT MICT	15 13	45–85 (range)	10	spinning bikes	fitness studio/S	HIIT: 10× 1 min @ 94% HR_max_, divided by 1 min low-intensity intervals; total session duration: not specified, 3 sessions/week MICT: a total of 30–50 min @ 81% HR_max_; 3 sessions/week
Knappett et al. [33]	male and female firefighters	HIIT MICT	8 7	43.3 ± 8.7 39.0 ± 5.0	4	rowing ergometer	workplace (fire station)/US	HIIT-1: 10× 20 s all-out sprints, divided by 40 s active recovery; total session duration: 14 min; 2 sessions/week MICT: a total of 25 min @ 65% HR_max_; 2 sessions/week
Kv et al. [49]	healthy males and females	HIIT CON	20 20	20.7 ± 1.8 20.8 ± 1.7	12	Tabata exercises	indoor stadium/US	HIIT: 8× 4min @ 75–80 HR_max_, divided by 1 min rest; total session duration: 30 min; 3 sessions/week CON: no exercise
Lunt et al. [34]	Inactive overweight males and females	HIIT-1 HIIT-2 MICT	16 16 17	48.2 ± 5.6 50.3 ± 8.0 46.3 ± 5.4	12	HIIT: walking/ jogging MICT: walking	outdoor (public park)/S	HIIT-1: 4× 4 min @ 90% HR_max_, divided by 3 min active recovery; total session duration: 40 min; 3 sessions/week HIIT-2: 3× 30 s all-out, divided by 4 min active recovery; total session duration: 25 min; 3 sessions/week MICT: a total of 48 min @ 60% HR_max_, 3 sessions/week
Musa et al. [50]	Untrained men	HIIT CON	23 22	29.8 ± 4.5 29.4 ± 4.9	8	running	sports complex track/US	HIIT: 4× 800 m @ 90% HR_max_, 1:1 work/rest ratio for 40 min; total session duration: 50 min; 3 sessions/week CON: no exercise
Ndlomo et al. [35]	sub-elite male soccer players	HIIT	18	21.0 ± 1.8	4	shuttle runs	outdoor (soccer field)/US	2 sets of 6–8× 6–15 s @ 120–170% vVO_2max_, divided by 9 s rest; total session duration: not specified; 2 sessions/week
Østerås et al. [36]	Healthy older untrained men and women	HIIT CON	13 11	68.9 ± 2.8 70.5 ± 3.0	10	running	outdoor/S	HIIT: 4× 4 min @ 90% HR_max_, divided by 4 min active recovery; total session duration: 60 min; 3 sessions/week CON: no training
Pérez-Ifrán et al. [51]	physically active males and females	HIIT-1 HIIT-2 CON	15 15 13	27 ± 5 (mean)	3	HIIT-1: running HIIT-2: burpees	indoor basketball court/US	Both HIIT protocols: 10× 4 s all-out, divided by 30 s recovery; total session duration: ~5 min; 5 sessions/week CON: no intervention
Prieur et al. [37] ^a^	Untrained men	HIIT-1	11	19.7 ± 1.7	6	running	outdoor/US	3 sets 6× 30 s all, divided by 30 s active rest; 6 min rest between sets; total session duration: 43.5 min; 3 sessions/week
Reljic et al. [38]	Sedentary male and female office workers	HIIT	109	48 ± 10 (mean)	26	spinning bikes	Workplace (indoor training room)/S	5× 1 min @ 85–95% HR_max_, divided by 1 min active recovery; total session duration: 14 min; 2 sessions/week
Reljic et al. [39]	Sedentary men and women	HIIT-1 HIIT-2 MICT	12 12 12	29.2 ± 6.0 29.9 ± 7.2 32.8 ± 8.4	8	spinning bikes	community-based fitness centre/S	HIIT-1: 5× 1 min @ 85–95% HR_max_, divided by 1 min active recovery; total session duration: 14 min; 2 sessions/week HIIT-2: 2× 4 min @ 85–95% HR_max_, divided by 2 min active recovery; total session duration: 15 min; 2 sessions/week MICT: 33 min @ 65–75% HR_max_; total session duration: 38 min; 2 sessions/week
Roy et al. [40]	overweight/obese males and females	HIIT MICT	104 146	43.5 ± 10.2 43.9 ± 11.5	52	Various exercise types	home, outdoor/US	HIIT: Various protocols (e.g., 10× 1 min) @ 80–90% HR_max_; total session duration: not specified; 3 sessions/week MICT: a total of 30 min @ moderate intensity; 5 sessions/week
Saadatnia et al. [41]	Untrained men	HIIT CON	13 10	23.3 ± 2.6 (mean)	10	running	outdoor/US	HIIT: 4–8× 30 s all-out, divided by 90 s passive recovery; total session duration: 20.5 min; 3 sessions/week CON: no training
Sandvei et al. [42]	sedentary to moderately trained men and women	HIIT MICT	11 12	25.2 ± 0.7 (mean)	8	HIIT: sprinting MICT: running	outdoor terrain/PS	HIIT: 5–10× 30 s sprints, divided by 3 min rest; total session duration: ~30–53 min; 3 sessions/week MICT: 30–60 min @ 70–80% HR_max_; total session duration: 45–75 min; 3 sessions/week
Scoubeau et al. [43]	Insufficiently physically active males and females	HIIT CON	14 14	23.1 ± 1.3 24.0 ± 3.9	8	total body exercises	home/US	HIIT: 4× 30 s all-out, divided by 30 s active recovery; total session duration: not specified; 3 sessions/week CON: remained inactive
Shepherd et al. [44]	Inactive males and females	HIIT MICT	46 46	42 ± 11 43 ± 11	10	HIIT: spinning bikes MICT: spinning, walking, jogging	community gym/S	HIIT: repeated 15–60 s bouts @ ~91% HR_max_, divided by 45–120 s recovery; total session duration: 18–25 min; 3 sessions/week MICT: a total of 30–45 min @ 70% HR_max_, 3 sessions/week plus 2 unsupervised moderate-intensity exercise sessions
Simonsson et al. [45]	regularly physically active males and females	HIIT MIT	34 34	69.7 ± 3.2 69.6 ± 2.8	12	indoor bicycle	gym/US	HIIT: 10× 6 s all-out, divide by 54 s recovery; total session duration: 20 min; 2 sessions/week MIT: 3× 8 min @ ~70% HRR, divided by 30 s recovery; total session duration: 40 min; 2 sessions/week
Song et al. [46]	male taekwondo athletes	HIIT-1 HIIT-2 CON	10 10 10	19.8 ± 1.3 (mean)	6	HIIT-1: repeated kicks HIIT-2: sprints CON: Taekwondo training	indoor training room/US	Both HIIT protocols: Taekwondo training + 3 sets of 10× 4 s all-out, divided by 15 s passive recovery; total session duration: 69 min; 3 sessions/week CON: Taekwondo training
Yan-guang et al. [52]	amphetamine-type stimulant-dependent males	HIIT MICT	40 46	33.7 ± 4.3 32.2 ± 5.1	52	HIIT: combined exercise types MICT: Tai Chi, mind–body exercise	basketball field, indoor fitness gym/S	HIIT: 26× 1 min @ 80–85% HR_max_, divided by 30 s recovery; not specified; total session duration: 60 min; 3 sessions/week MICT: a total of 60 min @ 55–65% HR_max_; 3 sessions/week

Notes: Total session duration values include warm-up, recovery phases between interval bouts and cool-down periods as reported by the original authors. Abbreviations: HIIT = High-intensity interval training; SIT = Sprint interval training; MICT = Moderate-intensity continuous training; MIT = Moderate-intensity training; CON = Control group; HR_max_ = Maximum heart rate; HRR = Heart rate reserve; VO_2max_ = Maximum oxygen uptake; VO_2peak_ = Peak oxygen uptake; V_max_ = Maximum running velocity; S = Supervised; PS = Partially supervised; US = Unsupervised.

For VO_2max/peak_, visual inspection of funnel plots suggested potential publication bias, supported by a significant Egger’s test result (*p* < 0.001; Figure S1). In contrast, neither visual interpretation of funnel plots nor Egger’s tests showed evidence of publication bias for SBP (*p* = 0.361; Figure S2A), DBP (*p* = 0.944; Figure S2B), TC (*p* = 0.916; Figure S2C), triglycerides (*p* = 0.805; Figure S2E), and WC (*p* = 0.798; Figure S2F). Funnel plot inspection suggested potential publication bias for LDL (Figure S2D), but Egger’s test indicated no significant statistical bias (*p* = 0.206). For all outcomes, sensitivity analyses showed no change in the significance or direction of the pooled effect sizes (Figures S3 and S4).

#### 3.4.2. Subgroup Analyses

Subgroup analyses based on participant type revealed significant moderating effects for SBP (Q = 17.8, df = 2, *p* < 0.001), DBP (Q = 14.9, df = 2, *p* = 0.001), body weight (Q = 20.8, df = 2, *p* < 0.001), and WC (Q = 14.1, df = 2, *p* = 0.001). Specifically, SBP (−6 mmHg; 95% CI: −8 to −5 mmHg, *p* < 0.001, k = 8) and DBP (−5 mmHg; 95% CI: −7 to −3 mmHg, *p* < 0.001, k = 8) only decreased significantly in overweight/clinical cohorts and healthy untrained cohorts (SBP: −3 mmHg; 95% CI: −5 to −1 mmHg, *p* = 0.006, k = 8; DBP: −2 mmHg; 95% CI: −3 to −1 mmHg, *p* = 0.044, k = 8) but not in healthy trained participants (SBP: −1 mmHg; 95% CI: −3 to 1 mmHg, *p* = 0.192, k = 3; DBP: −1 mmHg; 95% CI: −3 to 1 mmHg, *p* = 0.233, k = 3). Similarly, significant reductions in body weight (−4 kg; 95% CI: −6 to −3 kg, *p* < 0.001, k = 5) and WC (−3.4 cm; 95% CI: −5.4 to −1.4 cm, *p* = 0.001, k = 7) were only found in overweight/clinical cohorts but not in healthy untrained cohorts (body weight: 0.4 kg; 95% CI: −1.0 to 1.8 kg, *p* = 0.554, k = 9; WC: 0.9 cm; 95% CI: −1.7 to 1.9 cm, *p* = 0.925, k = 5) and healthy trained participants (body weight: −0.2 kg; 95% CI: −1.3 to 1.0 kg, *p* = 0.779, k = 7; WC: −0.3 cm; 95% CI: −2.0 to 1.4 cm, *p* = 0.738, k = 3).

Exercise modality significantly moderated WC changes (Q = 9.7, df = 1, *p* < 0.046). Subgroup analysis indicated that walking-/running-based HIIT elicited larger WC reductions (−3.6 cm; 95% CI: −5.3 to −1.7 cm, *p* = 0.001, k = 7) compared to cycling-based HIIT (0.2 cm; 95% CI: −2.0 to 2.3 cm, *p* = 0.876, k = 4). Furthermore, significant moderating effects of exercise setting were detected for DBP (Q = 8.5, df = 2, *p* = 0.014), HDL (Q = 6.3, df = 2, *p* = 0.043), and WC (Q = 7.7, df = 2, *p* = 0.022). Specifically, DBP and WC only decreased significantly in outdoor settings (−3 mmHg; 95% CI: −5 to −2 mmHg, *p* < 0.001, k = 4; and, −2.5 cm; 95% CI: −4.5 to −0.5 cm, *p* = 0.016, k = 3; respectively) and workplace/home-based settings (−4 mmHg; 95% CI: −5 to −3 mmHg, *p* < 0.001, k = 7; and, −2.9 cm; 95% CI: −3.9 to −1.9 cm, *p* = 0.010, k = 7; respectively) but not in gym-based settings (−1 mmHg; 95% CI: −2 to 0 mmHg, *p* = 0.083, k = 8; and, 0.2 cm; 95% CI: −1.2 to 1.6 cm, *p* = 0.762, k = 5; respectively). In contrast, gym-based settings were associated with a significant increase in HDL (4 mg/dL; 95% CI: 1 to 7 mg/dL, *p* = 0.020, k = 4), while outdoor (0 mg/dL; 95% CI: −2 to 1 mg/dL, *p* = 0.359, k = 5) and workplace/home-based settings (0 mg/dL; 95% CI: −2.8 to 2.3 mg/dL, *p* = 0.837, k = 6) showed no significant change.

Supervision status did not significantly influence the pooled pre–post changes in any outcome. For VO_2max/peak_, there was a descriptive trend toward a greater increase in supervised (4.1 mL/kg/min; 95% CI: 3.1 to 5.1 mL/kg/min, *p* < 0.001, k = 13) versus unsupervised settings (2.5 mL/kg/min; 95% CI: 1.2 to 3.7 mL/kg/min, *p* = 0.002, k = 17). However, these subgroup differences were not statistically significant (*p* = 0.091). No further significant moderating effects were observed for any of the analyzed outcomes.

#### 3.4.3. Meta-Regressions

Meta-regression analyses identified significant associations between exercise volume (min/week) and reductions in FG (β = −0.144, 95% CI: −0.277 to −0.011, *p* = 0.034) and SBP (β = −0.032, 95% CI: −0.059 to −0.001, *p* = 0.017). Moreover, longer intervention duration (study weeks) predicted larger decreases in BMI (β = −0.272, 95% CI: −0.464 to −0.080, *p* = 0.006) and DBP (β = −0.098, 95% CI: −0.173 to −0.024, *p* = 0.009). Meta-regression analyses did not reveal significant associations between intervention effects and weekly exercise volume or intervention duration for the remaining outcomes. Participants’ baseline age and weekly session frequency were not associated with changes in any outcome.

#### 3.4.4. HIIT Versus Passive Controls

**Figure 4:**
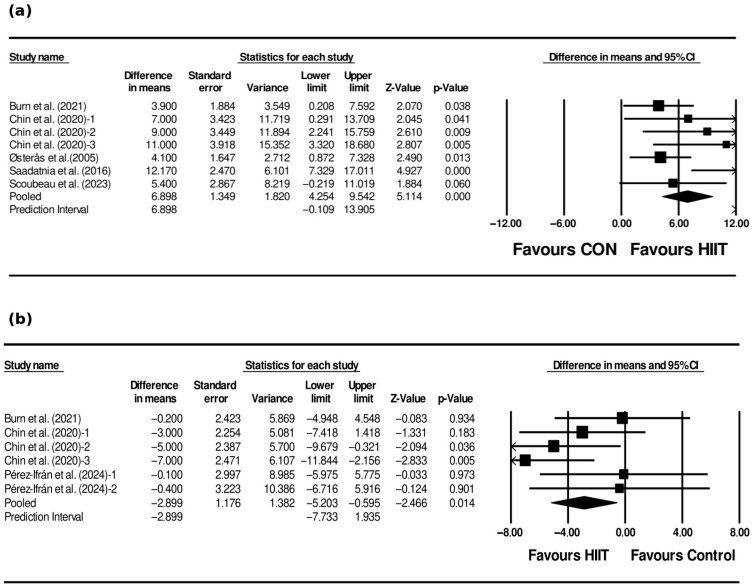
Forest plot of real-world HIIT effects versus passive controls on (**a**) VO_2max/peak_, and (**b**) waist circumference. Weighted mean differences and 95% CI confidence intervals. Squares represent the individual study effect estimates, horizontal lines represent the 95% confidence intervals, and diamonds represent the pooled effect estimates. Arrows indicate confidence intervals extending beyond the displayed axis range [26,27,36,41,43,51].

Compared with passive control conditions, HIIT resulted in a greater increase in VO_2max/peak_ (6.9 mL/kg/min; 95% CI: 4.3 to 9.5 mL/kg/min, *p* < 0.001; *I*^2^ *=* 47%; k = 7; Figure 4a). Moreover, HIIT led to a larger reduction in WC (−2.9 cm; 95% CI: −5.2 to −0.6 cm, *p* = 0.014; *I*^2^ = 20%; k = 6; Figure 4b) compared to passive controls. No statistically significant differences were observed for SBP (−3 mmHg; 95% CI: −10 to 3 mmHg, *p* = 0.314; *I*^2^ = 78%; k = 6) and DBP (−2 mmHg; 95% CI: −6 to 2 mmHg, *p* = 0.356; *I*^2^ = 72%; k = 6). Similarly, no statistically significant differences were found for FG (2 mg/dL; 95% CI: −2 to 6 mg/dL, *p* = 0.342; *I*^2^ = 51%; k = 4), triglycerides (−18 mg/dL; 95% CI: −42 to 5 mg/dL, *p* = 0.130; *I*^2^ = 40%; k = 4), TC (−5 mg/dL; 95% CI: −11 to 1 mg/dL, *p* = 0.104; *I*^2^ = 0%; k = 2), body weight (−1.0 kg; 95% CI: −5.1 to 3.1 kg, *p* = 0.641; *I*^2^ = 67%; k = 8), BMI (−0.8 kg/m^2^; 95% CI: −2.5 to 0.9 kg/m^2^, *p* = 0.370; *I*^2^ = 79%; k = 8), and BF% (−1.3%; 95% CI: −2.7 to−0.2%, *p* = 0.098; *I*^2^ = 0%; k = 7). Given an insufficient number of available studies, no pooled analyses were performed for HDL, LDL, insulin, and HOMA-IR.

For VO_2max/peak_ the funnel plot showed some visual asymmetry (Figure S5A), with studies predominantly favoring HIIT. However, Egger’s test indicated no statistically significant publication bias (*p* = 0.115). For WC, both the funnel plot (Figure S5B) and Egger’s test (*p* = 0.338) suggested an absence of significant publication bias. Sensitivity analyses did not affect the significance or direction of the pooled differences (Figure S6).

#### 3.4.5. HIIT Versus Active Controls

**Figure 5:**
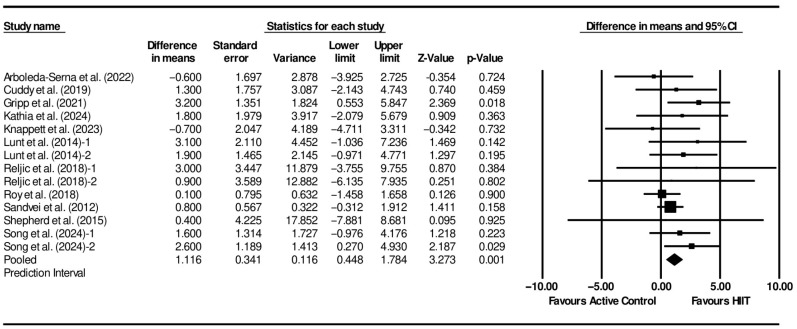
Forest plot of real-world HIIT effects versus active controls on VO_2max/peak_. Weighted mean differences and 95% confidence intervals. Squares represent the individual study effect estimates, horizontal lines represent the 95% confidence intervals, and diamonds represent the pooled effect estimates. Arrows indicate confidence intervals extending beyond the displayed axis range [23,28,30,32,33,34,39,40,42,44,46].

Significantly greater increases in VO_2max/peak_ were found in HIIT compared with active controls (1.1 mL/kg/min; 95% CI: 0.5 to 1.8 mL/kg/min, *p* = 0.001; *I*^2^ = 0%; k = 14; Figure 5). No differences were observed for SBP (−1 mmHg; 95% CI: −3 to 1 mmHg, *p* = 0.326; *I*^2^ = 0%; k = 8), DBP (0 mmHg; 95% CI: −2 to 2 mmHg, *p* = 0.919; *I*^2^ = 35%; k = 11), FG (−2 mg/dL; 95% CI: −5 to 1 mg/dL, *p* = 0.137; *I*^2^ = 0%; k = 5), TC (−4 mg/dL; 95% CI: −11 to 2 mg/dL, *p* = 0.175; *I*^2^ = 0%; k = 7), HDL (−2 mg/dL; 95% CI: −3 to 0 mg/dL, *p* = 0.111; *I*^2^ = 5%; k = 8), LDL (−6 mg/dL; 95% CI: −12 to 1 mg/dL, *p* = 0.104; *I*^2^ = 0%; k = 5), and triglycerides (−8 mg/dL; 95% CI: −20 to 5 mg/dL, *p* = 0.241; *I*^2^ = 33%; k = 7). Similarly, no differences were detected for body weight (−0.3 kg; 95% CI: −3.1 to 2.5 kg, *p* = 0.859; *I*^2^ = 0%; k = 6), BMI (−0.1 kg/m^2^; 95% CI: −0.9 to 0.8 kg/m^2^, *p* = 0.754; *I*^2^ = 0%; k = 8), BF% (−0.2%; 95% CI: −1.2 to 0.7%, *p* = 0.622; *I*^2^ = 0%; k = 10), and WC (−0.1 cm; 95% CI: −1.6 to 1.5 cm, *p* = 0.918; *I*^2^ = 0%; k = 7). Notably, the number of available studies was insufficient to permit pooled analyses for insulin and HOMA-IR in HIIT versus active control comparisons.

Visual inspection of the funnel plot for VO_2max/peak_ comparing HIIT to active control groups revealed a symmetrical distribution of study effects (Figure S7). This observation was corroborated by the Egger test, which showed no evidence of significant publication bias (*p* = 0.530). Moreover, the significance and direction of the pooled effect sizes remained unchanged in sensitivity analysis (Figure S8).

## 4. Discussion

To our knowledge, this meta-analysis is the first to examine the effectiveness of HIIT interventions implemented in real-world settings on CRF and cardiometabolic health in adults. Across 31 studies involving more than 1100 participants, the main findings indicate that real-world HIIT significantly improves CRF, as reflected by increases in VO_2max/peak_ across all analytic streams including controlled comparisons. For cardiometabolic outcomes, the evidence is less uniform. Several markers showed significant within-group improvements in pooled pre–post analyses, but the controlled evidence base is more limited in scope, statistical consistency, and number of contributing studies. Specifically, controlled comparisons demonstrate HIIT superiority over passive conditions only for VO_2max/peak_ and WC, while no cardiometabolic outcome change was superior to active control conditions. These distinctions are important for interpretation and are discussed in detail below.

### 4.1. Effects of Real-World HIIT: Pooled Pre–Post Analyses

It was a key result of our analyses that real-world HIIT resulted in significant improvements in CRF across a wide range of populations and settings. The observed pooled increase in VO_2max/peak_ of 3.9 mL/kg/min falls within the range reported in previous meta-analyses [7,53,54,55,56], suggesting that CRF improvements remain preserved when HIIT is translated into real-world practice. From a clinical and public-health perspective, this observation is important because higher CRF levels are strongly associated with lower risk of morbidity and premature mortality [57,58]. Mechanistically, improvements in VO_2max/peak_ arise from integrated central and peripheral adaptations across the oxygen transport and utilization cascade [59]. HIIT appears particularly effective in eliciting these adaptations, as repeated exposure to (near-)maximal intensities imposes potent hemodynamic and metabolic stimuli [60]. Against this background, the significant pooled increase in VO_2max/peak_ observed in the present analysis strengthens the translational relevance of real-world HIIT as a time-efficient exercise strategy for improving cardiorespiratory health in different populations. However, as the pooled analyses do not include a comparator condition, it is important to note that they cannot be used to attribute change causally to the intervention. Effects may partly reflect uncontrolled factors operating over the intervention period. Accordingly, the pre–post estimates reported in this section should be interpreted as descriptive evidence of the magnitude of change associated with real-world HIIT participation rather than as controlled effectiveness evidence. The primary basis for causal conclusions regarding HIIT effectiveness is provided by the controlled comparative analyses reported in Section 4.2 and Section 4.3. Moreover, the magnitude of the pooled VO_2max/peak_ effect should be interpreted cautiously. Heterogeneity among studies was high, and funnel plot asymmetry together with a significant Egger’s test suggests possible small-study effects or publication bias. Specifically, smaller real-world studies may have preferentially enrolled higher-motivated participants, implemented more intensive supervision, or been more likely to be published when outcomes were favorable. Thus, while the direction of effect appears robust, the pooled estimate may modestly overstate the magnitude of benefit expected in broader community implementation. Further, it should be noted that the included studies encompassed a heterogeneous mix of participant backgrounds, ranging from sedentary and clinical populations to recreationally active individuals and trained cohorts. These groups differ fundamentally in baseline CRF, training history, and cardiometabolic risk profile, and their pooled inclusion warrants explicit consideration.

Beyond CRF, pre–post analyses indicated within-group improvements in several cardiometabolic outcomes, including blood pressure, selected blood lipid markers, and body composition indices, though the strength and consistency of this evidence varies considerably across outcomes. This pattern is consistent with recent meta-analyses [8,11,53]. Notably, cardiometabolic outcomes also exhibited moderate-to-high heterogeneity. Specifically, exploratory subgroup analyses suggested greater blood pressure reductions in clinical and overweight/obese populations than in healthy trained individuals, a pattern consistent with the principle of baseline-dependent responsiveness. These findings are hypothesis-generating and should not be interpreted as establishing that participant type causally determines outcome magnitude, given the observational nature of subgroup comparisons, limited k values per subgroup cell, and the potential for residual confounding by correlated study characteristics such as intervention duration and supervision. Nevertheless, the direction of the pattern—larger cardiometabolic responsiveness in higher-risk populations—is biologically plausible and may inform targeted implementation of real-world HIIT.

Further, the findings on blood lipid profile alterations warrant a more nuanced and cautious interpretation. The significant reductions in TC, LDL, and triglycerides are physiologically plausible. It is well established that triglyceride concentrations respond sensitively to exercise-induced elevations in lipoprotein lipase activity and enhanced post-exercise lipid oxidation [61], while TC and LDL reductions likely reflect improved lipid turnover and favorable hepatic adaptations [62]. However, lipid outcomes are strongly confounded by diet, body composition, and baseline lipidemic status. As caloric and macronutrient intake were not stringently controlled across most studies, the isolated contribution of HIIT to lipid modulation cannot be established with absolute certainty.

Additionally, the limited number of studies reporting lipid outcomes increases susceptibility to numerical instability, despite the relatively low level of heterogeneity. These findings should therefore be regarded as preliminary signals warranting confirmation in future studies with larger evidence bases, rather than established effectiveness evidence.

Notably, significant reductions in BF%, and WC were observed despite no significant change in body weight. This finding appears contradictory at first glance. However, research has demonstrated that body weight alone can be a relatively insensitive metric, as it fails to differentiate between changes in fat mass and lean soft tissue, potentially remaining static despite meaningful body composition remodeling [63]. BF% and WC provide more granular insights into metabolically harmful adiposity [64,65] and the observed WC reduction is of particular clinical significance, as central adiposity serves as a more robust predictor of cardiometabolic risk than body weight or BMI [64]. Mechanistically, the metabolic demand of repeated high-intensity intervals may augment excess post-exercise oxygen consumption [66], stimulate catecholamine-mediated lipolysis, and optimize substrate partitioning [60,67], collectively promoting fat loss even when absolute reductions in body weight are marginal [53].

No significant pooled effects were found for FG, HDL, insulin, or HOMA-IR in any analytic stream, indicating that the current evidence base does not support conclusions regarding the glycemic or HDL-related effectiveness of real-world HIIT. These null findings should not necessarily be interpreted as evidence that real-world HIIT is ineffective for improving glycemic or insulin-related outcomes per se. More likely, they may reflect a combination of biological and methodological factors. Several included cohorts were healthy or active, limiting metabolic improvement potential, and study durations were often short. Notably, recent meta-analytic evidence suggests that HIIT interventions can significantly improve glycemic control in prediabetic and type 2 diabetic populations [8,9]. Additionally, as glycemic outcomes were less frequently reported than other parameters, beneficial effects may have been diluted when pooling across heterogeneous populations.

Exploratory subgroup analyses additionally suggested that exercise modality and setting may influence the pattern of distinctive cardiometabolic adaptations, though these findings must be interpreted with considerable caution. Walking-/running-based HIIT was associated with greater WC reductions than cycling-based protocols, which could plausibly reflect differences in whole-body muscle mass involvement and total energy turnover [67], but the subgroup comparison rested on small k values and may be confounded by systematic differences in participant characteristics or intervention design across modality groups. Similarly, outdoor and workplace/home-based settings showed nominally greater consistency in some cardiometabolic outcomes than gym-based settings, which may reflect ecological fit, lower implementation barriers, or differences in participant motivation and adherence—but again, these interpretations are speculative. The setting subgroup analyses draw on as few as 3–8 studies per cell, are conducted on pooled pre–post data without control conditions, and cannot account for the many correlated factors that differ between implementation contexts. These findings should therefore be regarded as hypothesis-generating observations that may guide the design of future research rather than evidence that specific settings or modalities causally determine cardiometabolic outcomes.

Meta-regression analyses identified associations between exercise volume and reductions in FG and SBP, and between longer intervention duration and decreases in BMI and DBP. These associations are directionally consistent with an established exercise dose–response relationship [68] and provide tentative support for the notion that exercise exposure may modulate the magnitude of selected cardiometabolic adaptations in real-world HIIT. However, these findings must also be interpreted as exploratory. Meta-regressions conducted on study-level aggregate data are subject to ecological fallacy, cannot establish within-individual dose–response relationships, and are susceptible to confounding by other study-level characteristics that correlate with exercise volume or duration. The number of effect sizes available for several outcomes was at or near the minimum threshold for meta-regression (k ≥ 10), limiting statistical reliability. In the case of FG, the significant volume-response association should be interpreted particularly cautiously given the null overall pooled effect. These associations therefore generate hypotheses for future individual-level dose–response analyses rather than supporting prescriptive conclusions about optimal training parameters.

Taken together, the pre–post analyses provide a consistent signal of some within-group cardiometabolic changes associated with real-world HIIT participation across diverse settings and populations. However, it is important to note that the majority of these significant pre–post effects are not corroborated by the controlled comparative analyses, in which only WC reached significance versus passive controls and no cardiometabolic outcome was significant versus active controls. Accordingly, these pre–post findings should be regarded as hypothesis-generating rather than conclusive effectiveness evidence, and their interpretation should be tempered by the inferential limitations inherent to uncontrolled within-group analyses.

### 4.2. HIIT Versus Passive Controls

Comparisons with passive controls provide a direct estimate of real-world HIIT effectiveness relative to non-exercise behavior. The most robust finding was a substantially greater VO_2max/peak_ improvement with HIIT by 6.9 mL/kg/min. The magnitude of this effect, together with moderate heterogeneity, indicates a relatively consistent advantage of HIIT over non-exercise conditions across real-world settings. This is in line with extensive evidence for HIIT-induced CRF gains versus controls [7,53,54,55,56]. HIIT also produced significantly greater WC reductions than passive controls, which is consistent with prior research [53,67].

No significant differences were observed for other cardiometabolic outcomes, though these null findings warrant cautious interpretation. First, the number of studies contributing to these analyses was limited, reducing statistical power. Second, substantial heterogeneity was observed for several outcomes, particularly blood pressure. Third, several interventions were relatively short in duration and may not have been sufficiently powered to detect significant changes in slower-adapting cardiometabolic markers. Moreover, there were insufficient studies to perform pooled comparative analyses for several outcomes, reflecting a broader pattern in real-world studies, where fitness and anthropometric outcomes are reported more consistently than metabolic biomarkers. Null findings may therefore reflect data limitations rather than true absence of effect.

### 4.3. HIIT Versus Active Controls

Compared to other structured exercise modalities, HIIT provided a modest but consistent VO_2max/peak_ advantage, while no significant superiority was observed for cardiometabolic risk markers or body composition. The CRF advantage supports the established notion that interval-based exercise provides a potent cardiorespiratory stimulus [60] consistent with prior meta-analyses showing superior and more time-efficient CRF gains with HIIT compared with MICT [7,55,69]. Importantly, negligible heterogeneity and no publication bias suggest this advantage is both robust and generalizable to real-world settings.

The absence of significant differences for cardiometabolic outcomes suggests HIIT and other structured exercise modalities yield broadly comparable benefits in real-world settings. This finding is consistent with prior meta-analyses [55,70] indicating that cardiometabolic adaptations may be primarily driven by total energy expenditure and exercise volume.

### 4.4. Practical Implications

The present findings have several implications for clinicians, exercise professionals, workplace health providers, and public-health stakeholders. First, HIIT can be considered a viable and effective real-world exercise strategy for improving CRF across a range of populations and settings. Given that lack of time is one of the most commonly reported barriers to physical activity, the time-efficient nature of HIIT may enhance feasibility and uptake, particularly in workplace, community, and preventive health contexts. The modest but consistent superiority of HIIT for VO_2max/peak_ gains compared with active control interventions may be relevant in settings, where improving CRF is a primary objective, such as rehabilitation, preventive cardiology, and occupational health. Second, real-world HIIT appears to confer additional benefits for selected cardiometabolic outcomes, including blood pressure, central adiposity, and lipid profiles, particularly in individuals with elevated baseline risk. Accordingly, HIIT may be appropriately integrated into lifestyle interventions targeting cardiometabolic health. However, the current evidence does not support positioning HIIT as universally superior to other exercise modalities. When compared with active interventions such as MICT, most cardiometabolic benefits appear broadly comparable. Therefore, exercise prescription should remain individualized and guided by patient preference, clinical status, and feasibility. Within this framework, HIIT should be viewed as an effective, time-efficient and well-accepted [71] option within a broader continuum of exercise strategies rather than a universally superior approach.

### 4.5. Limitations and Future Directions

Several limitations warrant consideration when interpreting our findings. First, substantial heterogeneity across pooled analyses reflects genuine variability in participant characteristics, intervention design, and settings. Pooled estimates should therefore be interpreted as average effects across heterogeneous implementations rather than precise effect sizes of a standardized intervention. Specifically, the inclusion of participants with widely differing baseline fitness levels—ranging from sedentary clinical populations to recreationally active adults and trained cohorts—represents both a strength and a limitation of the present review. On the one hand, it enhances the external validity of the findings by reflecting the diversity of individuals participating in real-world HIIT programs. On the other hand, physiological responsiveness differs substantially according to baseline fitness and cardiometabolic health, and the pooling of these groups inevitably increases between-study heterogeneity. Importantly, to avoid conflating fundamentally different populations, training status was prespecified as a subgroup moderator. These analyses demonstrated that several cardiometabolic benefits, including blood pressure, body weight, and WC reductions, were concentrated in overweight/clinical and previously untrained individuals, while trained participants showed smaller or non-significant responses, consistent with a ceiling effect in populations with already-favorable cardiometabolic profiles. The trained participant subgroup was relatively small (3–8 studies, depending on outcome), which limits the precision of these estimates and should be considered when interpreting null findings in this subgroup. Future reviews with a sufficient number of sport-specific studies may benefit from analyzing these populations separately to further characterize population-specific effectiveness.

Second, all subgroup and meta-regression analyses must be regarded as exploratory and hypothesis-generating. These analyses were conducted on aggregated study-level data, are subject to ecological bias and residual confounding, and involved subgroup cells with limited contributing studies. Multiple subgroup comparisons were performed across several outcomes and moderators, increasing the risk of false-positive findings. Accordingly, subgroup and meta-regression findings should not be used to draw prescriptive conclusions about which populations, settings, modalities, or training parameters determine HIIT effectiveness. Rather, they are intended to identify patterns that warrant investigation in future hypothesis-driven research.

Third, the overall certainty of evidence must be considered low to moderate, particularly for cardiometabolic outcomes. Several methodological features of the included studies collectively limit confidence in the pooled estimates. Blinding of participants was not feasible given the nature of exercise interventions, and assessor blinding was inconsistently reported. Sample sizes were small in many studies, reducing precision and increasing susceptibility to random error. Even among controlled trials, non-randomized designs were represented, limiting the strength of causal inference. Critically, six of the 31 included studies employed single-group pre–post designs without a control condition, contributing only uncontrolled within-group data to the pooled pre–post analyses. Accordingly, pre–post estimates should be regarded as descriptive evidence of within-group responsiveness under real-world conditions, while the comparative analyses—HIIT versus passive and active controls—represent the primary basis for effectiveness conclusions and should be weighted more heavily when drawing inferential conclusions. Furthermore, seven studies contributed multiple HIIT intervention arms to the analyses. Although unit-of-analysis procedures were applied to mitigate precision inflation in comparative analyses, the residual dependency between arms from the same study cannot be fully eliminated and may modestly underestimate true between-study variance. Taken together, these features suggest that the pooled estimates—while consistent in direction—should be interpreted with appropriate epistemic caution, and that firm conclusions about the magnitude of HIIT-induced cardiometabolic benefits in real-world settings await confirmation from larger, well-controlled trials. A related limitation in the comparative evidence concerns several cardiometabolic outcomes—specifically HDL, LDL, insulin, and HOMA-IR—for which an insufficient number of controlled studies precluded pooled comparative analyses entirely. Consequently, no comparative effectiveness conclusions can therefore be drawn for these outcomes, and their inclusion in future controlled real-world HIIT trials should be prioritized to close this evidentiary gap.

Fourth, publication bias cannot be excluded. Funnel plot asymmetry and a significant Egger’s test for VO_2max/peak_ suggest overrepresentation of larger effects, potentially inflating the magnitude of CRF improvements in both pre–post and comparative pooled estimates. Additionally, limited data for several cardiometabolic outcomes precluded comprehensive pooling, restricting firm conclusions regarding metabolic effects of real-world HIIT.

Fifth, an important limitation relates to the heterogeneous nature of real-world implementation itself. Rather than representing a binary concept, real-world implementation should be viewed as a continuum. Although all included interventions were conducted outside dedicated exercise physiology laboratories, they differed substantially with respect to supervision, participant autonomy, implementation fidelity, and delivery context. While some programs remained highly structured despite being delivered in community or workplace settings, others relied largely on independent participant execution. In particular, supervised programs conducted in workplace or community settings allow more standardized protocol delivery and fidelity monitoring, while unsupervised home-based or self-directed programs are inherently more variable in execution—a distinction with meaningful implications for reproducibility and practical scalability. The supervision subgroup analyses were specifically designed to quantify the extent to which this variation influences outcomes. The absence of a significant supervision effect across all analyzed outcomes provides empirical support for the pooled approach, while simultaneously identifying supervised delivery as associated with a descriptively larger CRF response—a finding that may be clinically meaningful despite falling short of statistical significance at the current sample sizes. Consequently, the present findings should be interpreted as reflecting the effectiveness of HIIT across a broad spectrum of pragmatic implementation models rather than a single, homogeneous real-world intervention. Although this diversity enhances ecological validity and reflects routine practice, it also contributes to between-study heterogeneity and limits the precision with which specific implementation models can be compared. Future effectiveness studies would benefit from standardized reporting of implementation characteristics to facilitate more refined comparisons across different real-world delivery contexts.

Sixth, key confounders were often inadequately controlled or reported in the primary studies, which weakens the internal validity of the cardiometabolic findings specifically. Dietary intake was rarely monitored or reported, yet blood lipids, FG, body weight, and blood pressure are all directly sensitive to dietary composition and caloric balance. Concurrent changes in diet during the intervention period—whether spontaneous or intervention-induced—may have contributed to or partially explained the observed cardiometabolic improvements, independently of the exercise stimulus. Similarly, medication use was infrequently documented. The initiation, modification, or discontinuation of lipid-lowering agents, antihypertensive medications, or hypoglycemic drugs during an intervention period could plausibly account for some of the observed changes in blood pressure, blood lipids, and FG, particularly in clinical and overweight populations where such medications are prevalent. Further, habitual physical activity outside the prescribed HIIT sessions was rarely assessed or controlled, making it impossible to determine whether participants changed their overall activity behavior during the intervention. Increases in habitual activity—a plausible accompaniment to participation in an exercise program—could independently contribute to cardiometabolic improvements beyond the HIIT sessions themselves. Collectively, these uncontrolled factors introduce meaningful confounding risk to the cardiometabolic effect estimates and preclude definitive causal attribution of the observed changes to the HIIT intervention in the absence of controlled comparative data. This limitation is most consequential for outcomes supported primarily by pre–post analyses—particularly blood lipids, FG, and body weight—and less consequential for VO_2max/peak_, where the controlled comparative analyses provide independent, confounding-resistant evidence of a real HIIT-specific effect.

Seventh, the method used to assess VO_2max_ or VO_2peak_ varied across included studies. While several studies employed direct incremental cardiopulmonary exercise testing with respiratory gas analysis—the criterion standard for CRF measurement—others used field-based estimation protocols such as the multistage shuttle run. Direct and estimated values are not interchangeable. Field-based estimates introduce additional measurement error and may systematically over- or underestimate true VO_2max/peak_ depending on the population, test administrator, and environmental conditions. Pooling these values as a common metric assumes a degree of measurement equivalence that is not always warranted and may contribute to the high between-study heterogeneity observed for VO_2max/peak_. Future real-world HIIT studies should prioritize direct VO_2max/peak_ assessment or, where this is not feasible, report the specific estimation protocol and its known measurement error to facilitate more comparable pooling across studies.

Finally, a potential limitation relates to the operational definition of HIIT. Contemporary HIIT encompasses a broad spectrum of protocols differing in exercise modality, interval duration, work-to-rest ratio, and prescribed intensity. Consequently, no universally accepted physiological threshold exists to distinguish HIIT from other vigorous interval exercise. To minimize subjectivity, eligibility was based on predefined structural characteristics of HIIT rather than study terminology alone. Nevertheless, some included functional or circuit-based interventions may differ from traditional endurance-based HIIT, which should be considered when interpreting the pooled findings.

Therefore, future research should address several priorities. Larger, adequately powered real-world RCTs are needed to confirm the effects of HIIT on CRF and cardiometabolic outcomes with sufficient duration to detect meaningful long-term adaptations. Greater standardization and reporting of adherence, intervention fidelity, and achieved exercise intensity are also needed, as real-world effectiveness depends on sustained exposure to the intended exercise stimulus. Future studies should also evaluate strategies to improve adherence, including digital support, behavioral interventions, and individualized program design. Given the potentially greater responsiveness of overweight and clinical cohorts, it should also be further explored how HIIT can be tailored to maximize benefits in higher-risk populations. Such approaches may help clarify for whom HIIT is most effective, under which conditions, and with what degree of sustainability and scalability as a public-health strategy.

## 5. Conclusions

Our findings indicate that real-world HIIT consistently and robustly improves CRF in adults, supported by both pre–post and controlled comparative evidence. For cardiometabolic outcomes, the picture is more nuanced. While pre–post analyses demonstrated significant within-group improvements in blood pressure, lipid markers, and body composition, these effects were largely not confirmed in controlled comparisons, where HIIT superiority over passive controls was limited to WC and no advantage over active exercise controls was observed for any cardiometabolic outcome. The interpretation of cardiometabolic findings is further constrained by inadequate control and reporting of key confounders—particularly diet, medication use, and habitual physical activity outside sessions—in the primary studies, which limits causal attribution of the observed changes to the HIIT intervention itself. CRF improvement therefore represents the most consistently supported and causally attributable benefit of real-world HIIT, while cardiometabolic effects—though biologically plausible—currently rest on a more limited controlled evidence base and warrant cautious interpretation. Taken together, these findings suggest that HIIT retains translational potential beyond laboratory settings, particularly as a time-efficient strategy for improving CRF. However, considerable heterogeneity, moderate methodological quality, and limited controlled evidence for several outcomes warrant cautious interpretation. Real-world HIIT is best regarded not as universally superior, but as an effective, scalable exercise option whose benefits are maximized when tailored to individual characteristics, context, and adherence.

## Data Availability

The datasets generated and analyzed during the current study are not publicly available but are available from the corresponding author on reasonable request.

## Supplementary Materials

The following supporting information can be downloaded at: https://www.mdpi.com/article/10.3390/sports14070311/s1, Table S1: Detailed search strategy; Table S2: Methodological quality assessment of included RCTs (Pedro Score); Table S3: Methodological quality assessment of included single-group studies (NIH assessment tool); Figure S1: Funnel plot for the effects of real-world HIIT on VO_2max/peak_; Figure S2: Funnel plots for the effects of real-world HIIT on (a) systolic blood pressure, (b) diastolic blood pressure, (c) total cholesterol, (d) LDL cholesterol, (e)’triglycerides, and (f) waist circumference; Figure S3: Forest plot of sensitivity analysis for the effects of real-world HIIT on VO_2max/peak_; Figure S4: Forest plots of sensitivity analysis for the effects of real-world HIIT on (a) systolic blood pressure, (b) diastolic blood pressure, (c) total cholesterol, (d) LDL cholesterol, (e) triglycerides, and (f) waist circumference; Figure S5: Funnel plots for the effects of real-world HIIT versus passive controls on (a) VO_2max/peak_, and (b) waist circumference; Figure S6. Forest plots of sensitivity analysis for the effects of real-world HIIT versus passive controls on (a) VO_2max_/_peak_, and (b) waist circumference; Figure S7: Funnel plot for the effects of real-world HIIT versus active controls on VO_2max/peak_; Figure S8: Forest plot of sensitivity analysis for the effects of real-world HIIT versus active controls on VO_2max/peak_.

## Abbreviations

The following abbreviations are used in this manuscript:

HIIT	High-intensity interval training
CRF	Cardiorespiratory fitness
WMD	Weighted mean differences
VO_2max_	Maximal oxygen uptake
LDL	Low-density lipoprotein cholesterol
SIT	Sprint interval training
MICT	Moderate-intensity continuous training
BMI	Body mass index
VO_2peak_	Peak oxygen uptake
SBP	Systolic blood pressure
DBP	Diastolic blood pressure
FG	Fasting glucose
TC	Total cholesterol H
HDL	High-density lipoprotein cholesterol
HOMA-IR	Homeo-stasis Model Assessment of Insulin Resistance
BF%	Body fat percentage
WC	Waist circumference
RCTs	Randomized controlled trials
CIs	Confidence intervals
HR_max_	Maximum heart rate
MIT	Moderate-intensity training
CON	Control group
HRR	Heart rate reserve
V_max_	Maximum running velocity
