# Supplementary material for: Effects of High-Intensity Interval Training on Cardiorespiratory Fitness and Cardiometabolic Health in Real-World Settings: A Systematic Review and Meta-Analysis
Source: Sports (Basel). 2026 Jul 22;14(7):311. doi: 10.3390/sports14070311 (PMC13416871; doi:10.3390/sports14070311)
Supplement: Supplementary file 1 [file sports-14-00311-s001.zip › sports-4389195-supplementary.pdf]

SUPPLEMENTARY MATERIALS

Table S1. Detailed search strategy.

**Database (total records)**	**Search terms**
Pubmed/MedLine (n = 968)	(("high-intensity interval training"[Title/Abstract] OR "HIIT"[Title/Abstract] OR "interval training"[Title/Abstract] OR "interval exercise"[Title/Abstract] OR "sprint interval training"[Title/Abstract]) AND ("real-world"[Title/Abstract] OR "real-life"[Title/Abstract] OR "real-world setting"[Title/Abstract] OR "real-life setting"[Title/Abstract] OR "community setting"[Title/Abstract] OR "community-based"[Title/Abstract] OR "workplace"[Title/Abstract] OR "worksite"[Title/Abstract] OR "home-based"[Title/Abstract] OR "outdoor"[Title/Abstract] OR "field-based"[Title/Abstract] OR "fitness center"[Title/Abstract] OR "fitness centre"[Title/Abstract] OR "gym"[Title/Abstract] OR "gym-setting"[Title/Abstract]))
SPORTDiscus (n = 5441)	("high-intensity interval training" OR "HIIT" OR "interval training" OR "interval exercise" OR "sprint interval training") AND ("real-world" OR "real-life" OR "real-world setting" OR "real-life setting" OR "community setting" OR "community-based" OR "workplace" OR "worksite" OR "home-based" OR "outdoor" OR "field-based" OR "fitness center" OR "fitness centre" OR "gym" OR "gym-setting")
Scopus (n = 51)	(TITLE-ABS-KEY("high-intensity interval training") OR TITLE-ABS-KEY("HIIT") OR TITLE-ABS-KEY("interval training") OR TITLE-ABS-KEY("interval exercise") OR TITLE-ABS-KEY("sprint interval training")) AND (TITLE-ABS-KEY("real-world") OR TITLE-ABS-KEY("real-life") OR TITLE-ABS-KEY("real-world setting") OR TITLE-ABS-KEY("real-life setting") OR TITLE-ABS-KEY("community setting") OR TITLE-ABS-KEY("community-based") OR TITLE-ABS-KEY("workplace") OR TITLE-ABS-KEY("worksite") OR TITLE-ABS-KEY("home-based") OR TITLE-ABS-KEY("outdoor") OR TITLE-ABS-KEY("field-based") OR TITLE-ABS-KEY("fitness center") OR TITLE-ABS-KEY("fitness centre") OR TITLE-ABS-KEY("gym") OR TITLE-ABS-KEY("gym-setting"))
Cochrane Central Register of Controlled Trials (CENTRAL) (n = 806)	("high-intensity interval training" OR "HIIT" OR "interval training" OR "interval exercise" OR "sprint interval training") AND ("real-world" OR "real-life" OR "real-world setting" OR "real-life setting" OR "community setting" OR "community-based" OR "workplace" OR "worksite" OR "home-based" OR "outdoor" OR "field-based" OR "fitness center" OR "fitness centre" OR "gym" OR "gym-setting")
Web of Science (n = 446)	TS=("high-intensity interval training" OR "HIIT" OR "interval training" OR "interval exercise" OR "sprint interval training") AND TS=("real-world" OR "real-life" OR "real-world setting" OR "real-life setting" OR "community setting" OR "community-based" OR "workplace" OR "worksite" OR "home-based" OR "outdoor" OR "field-based" OR "fitness center" OR "fitness centre" OR "gym" OR "gym-setting")

Notes: Searches were conducted in five electronic databases from inception to December 2025. Record counts reflect total records retrieved per database prior to de-duplication. Database-specific field tags and syntax are applied where indicated. OR connects synonyms within each conceptual block; AND links the “HIIT” block to the “real-world setting” block.

Table S2. Methodological quality assessment of included RCTs (PEDro Score).

Study	C-1	C-2	C-3	C-4	C-5	C-6	C-7	C-8	C-9	C-10	C-11	Pedro Score
Arboleda-Serna et al. [23]	Yes	1	1	1	0	0	0	1	1	1	1	7
Brown et al. [25]	Yes	1	1	1	0	0	0	1	1	1	1	7
Burn et al. [26]	Yes	0	0	1	0	0	0	1	1	1	1	3
Cebrick-Grossman et al. [27]	Yes	1	0	1	0	0	0	0	1	1	1	4
Chin et al. [28]	Yes	1	0	1	0	0	0	0	1	1	1	5
Cuddy et al. [29]	Yes	1	0	1	0	0	0	1	1	1	1	6
D'Alleva et al. [30]	Yes	1	1	1	0	0	0	1	1	1	1	7
Eather et al. [31]	Yes	1	0	1	0	0	1	0	1	1	1	6
Gripp et al [32]	Yes	1	0	1	0	0	0	0	1	1	1	5
Holmes et al. [33]	Yes	1	0	1	0	0	0	1	1	1	1	6
Kathia et al. [34]	Yes	1	1	1	0	0	1	1	1	1	1	8
Knappett et al. [35]	Yes	1	1	1	0	0	0	1	1	1	1	7
Kv et al. [36]	Yes	1	1	0	0	0	0	1	1	1	1	6
Lunt et al. [37]	Yes	1	1	1	0	0	1	0	1	1	1	7
Musa et al. [38]	Yes	1	0	1	0	0	0	0	1	1	1	5
Østerås et al. [40]	Yes	1	0	1	0	0	0	1	1	1	1	6
Pérez-Ifrán et al. [41]	Yes	1	0	1	0	0	0	0	1	1	1	5
Reljic et al. [44]	Yes	1	1	1	0	1	1	0	1	1	1	8
Saadatnia et al. [46]	Yes	1	0	1	0	0	0	1	1	1	1	6
Sandvei et al. [47]	Yes	1	0	1	0	0	0	0	1	1	1	5
Scoubeau et al. [48]	Yes	1	1	1	0	0	0	1	1	1	1	7
Shepherd et al. [49]	Yes	1	1	1	0	0	0	1	1	1	1	7
Simonsson et al. [50]	Yes	1	1	1	0	0	1	1	1	1	1	8
Song et al. [51]	Yes	1	0	1	0	0	0	1	1	1	1	6
Yan-Guang et al. [52]	Yes	1	1	1	0	0	1	0	1	1	1	7

Notes: C = criterion; 1 = eligibility criteria specified; 2 = random allocation of subjects; 3 = concealed allocation; 4 = similar groups at baseline; 5 = blinding of subjects; 6 = blinding of therapists; 7 = blinding of assessors; 8 = key outcome obtained from >85% of allocated subjects; 9 = intention to treat analysis; 10 = between-group comparisons for at least one key outcome; 11 = Point measures and measures of variability for at least one key outcome; Pedro Score = overall score.

Table S3. Methodological quality assessment of included single-group studies (NIH assessment tool).

Study	C-1	C-2	C-3	C-4	C-5	C-6	C-7	C-8	C-9	C-10	C-11	C-12
Allison et al. [22]	Yes	Yes	No	NR	No	Yes	Yes	NR	Yes	Yes	No	No
Bielec et al. [24]	Yes	No	No	NR	No	Yes	Yes	NR	Yes	Yes	No	No
Ndlomo et al. [39]	Yes	No	No	NR	No	Yes	Yes	NR	Yes	Yes	No	No
Prieur et al. [42]	Yes	No	No	NR	No	Yes	Yes	NR	Yes	Yes	No	No
Reljic et al. [43]	Yes	Yes	Yes	No	No	Yes	Yes	No	No	Yes	Yes	Yes
Roy et al. [45]	Yes	Yes	Yes	NR	No	Yes	Yes	Yes	No	Yes	Yes	Yes

Notes: C = criterion; 1 = clearly stated research question; 2 = eligibility criteria prespecified; 3 = representative sample; 4 = all eligible participants enrolled; 5 = sample size justification; 6 = intervention clearly described; 7 = outcomes clearly defined, valid, reliable; 8 = blinded outcome assessors; 9 = follow-up rate ≥80%; 10 = appropriate statistical analyses; 11 = multiple outcome measurements over time; 12 = confounding variables accounted for.

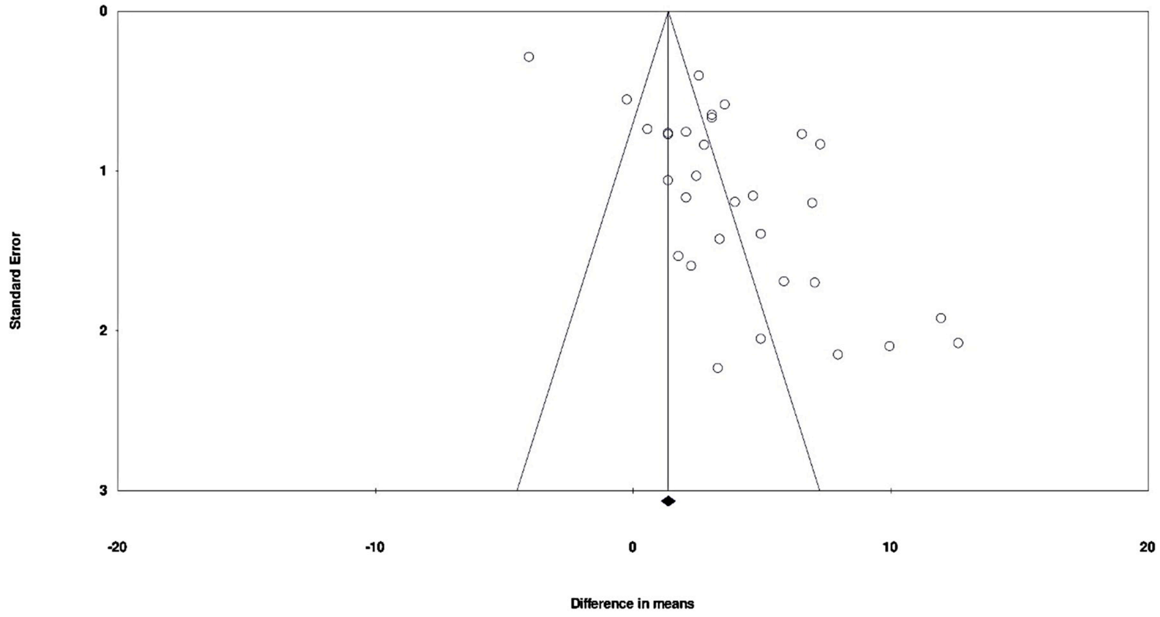

Figure S1. Funnel plot for the effects of real-world HIIT on VO_{2max}.

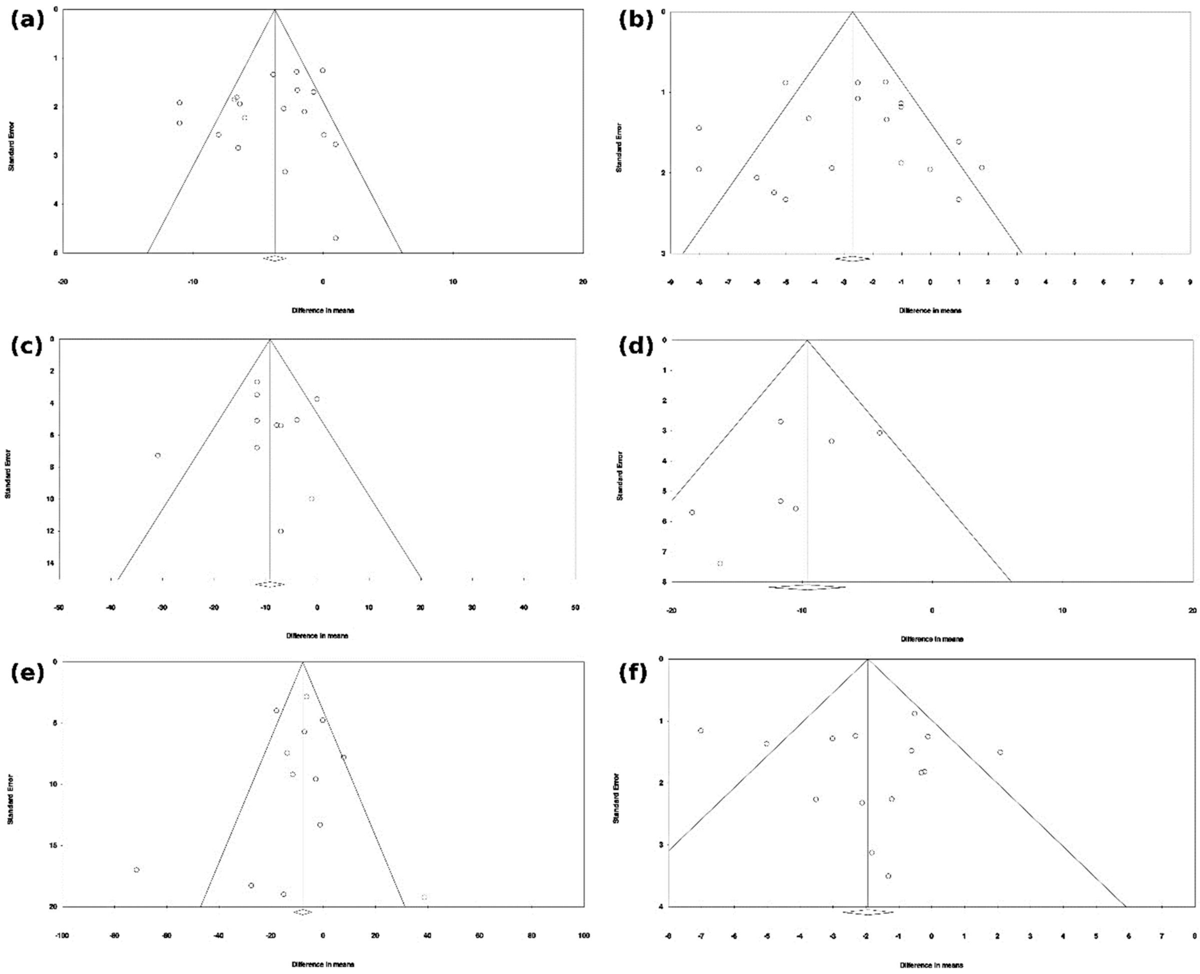

Figure S2. Funnel plots for the effects of real-world HIIT on (**a**) systolic blood pressure, (**b**) diastolic blood pressure, (**c)** total cholesterol, (**d**) LDL cholesterol, (**e**) triglycerides, and (f) waist circumference.

Study name	Statistics with study removed						
	Point	Standard error	Variance	Lower limit	Upper limit	Z-Value	p-Value
Allison et al. (2017)	3,938	0,711	0,505	2,546	5,331	5,542	0,000
Arboleda-Serna et al. (2022)	3,927	0,712	0,507	2,531	5,322	5,515	0,000
Bielec et al. (2017)	3,957	0,450	0,202	3,076	4,838	8,800	0,000
Brown et al. (2018)-1	3,950	0,709	0,502	2,561	5,339	5,573	0,000
Brown et al. (2018)-2	3,892	0,704	0,496	2,512	5,272	5,528	0,000
Burn et al. (2021)	3,851	0,707	0,499	2,466	5,236	5,450	0,000
Chin et al. (2020)-1	3,765	0,700	0,490	2,393	5,136	5,380	0,000
Chin et al. (2020)-2	3,707	0,696	0,484	2,343	5,071	5,327	0,000
Chin et al. (2020)-3	3,640	0,689	0,475	2,289	4,992	5,281	0,000
Cuddy et al. (2019)	3,911	0,720	0,518	2,501	5,322	5,436	0,000
D'Alleva et al. (2023)	3,772	0,699	0,488	2,402	5,141	5,398	0,000
Gripp et al. (2021)	3,749	0,689	0,474	2,399	5,099	5,444	0,000
Holmes et al. (2023)-1	3,875	0,708	0,501	2,487	5,262	5,473	0,000
Holmes et al. (2023)-2	3,842	0,705	0,497	2,460	5,224	5,449	0,000
Kathia et al. (2024)	3,894	0,707	0,500	2,507	5,280	5,504	0,000
Knappett et al. (2023)	3,927	0,707	0,500	2,541	5,313	5,553	0,000
Lunt et al. (2014)-1	3,943	0,708	0,501	2,556	5,330	5,572	0,000
Lunt et al. (2014)-2	3,998	0,719	0,518	2,588	5,408	5,557	0,000
Ndlomo et al. (2023)	3,776	0,693	0,480	2,417	5,134	5,447	0,000
Østerås et al.(2005)	3,894	0,720	0,519	2,483	5,306	5,407	0,000
Prieur et al. (2013)	3,963	0,712	0,507	2,567	5,359	5,565	0,000
Reljic et al. (2018)-1	3,780	0,701	0,491	2,406	5,153	5,394	0,000
Reljic et al. (2018)-2	3,846	0,703	0,495	2,468	5,225	5,468	0,000
Reljic et al. (2022)	3,970	0,719	0,517	2,561	5,378	5,523	0,000
Roy et al. (2018)	4,031	0,727	0,528	2,607	5,455	5,549	0,000
Saadatnia et al. (2016)	3,631	0,689	0,475	2,280	4,982	5,268	0,000
Sandvei et al. (2012)	3,942	0,746	0,557	2,479	5,405	5,281	0,000
Scoubeau et al. (2023)	3,816	0,703	0,494	2,438	5,194	5,429	0,000
Shepherd et al. (2015)	3,919	0,715	0,511	2,517	5,321	5,480	0,000
Simonsson et al. (2023)	3,970	0,719	0,517	2,560	5,379	5,521	0,000
Song et al. (2024)-1	3,945	0,719	0,517	2,537	5,354	5,489	0,000
Song et al. (2024)-2	3,912	0,720	0,519	2,500	5,323	5,431	0,000
Pooled	3,877	0,693	0,481	2,518	5,236	5,592	0,000
Prediction Interval	3,877	0,000	0,000	-3,822	11,575	0,000	0,000

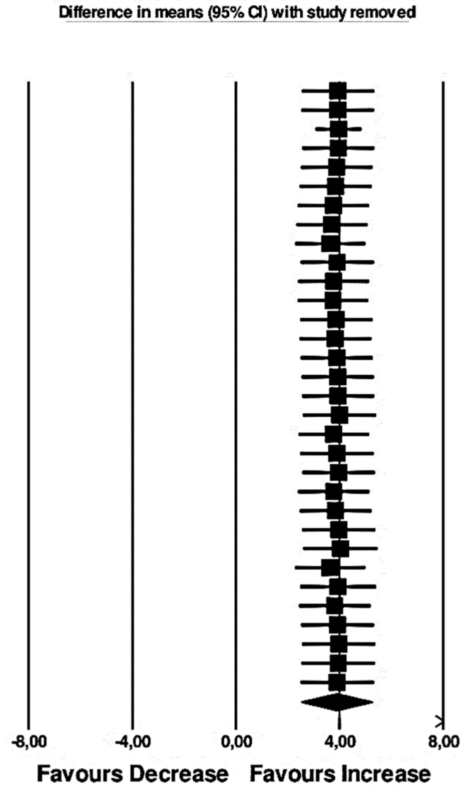

Figure S3. Forest plot of sensitivity analysis for the effects of real-world HIIT on VO_{2max}. Weighted mean differences and 95% confidence intervals

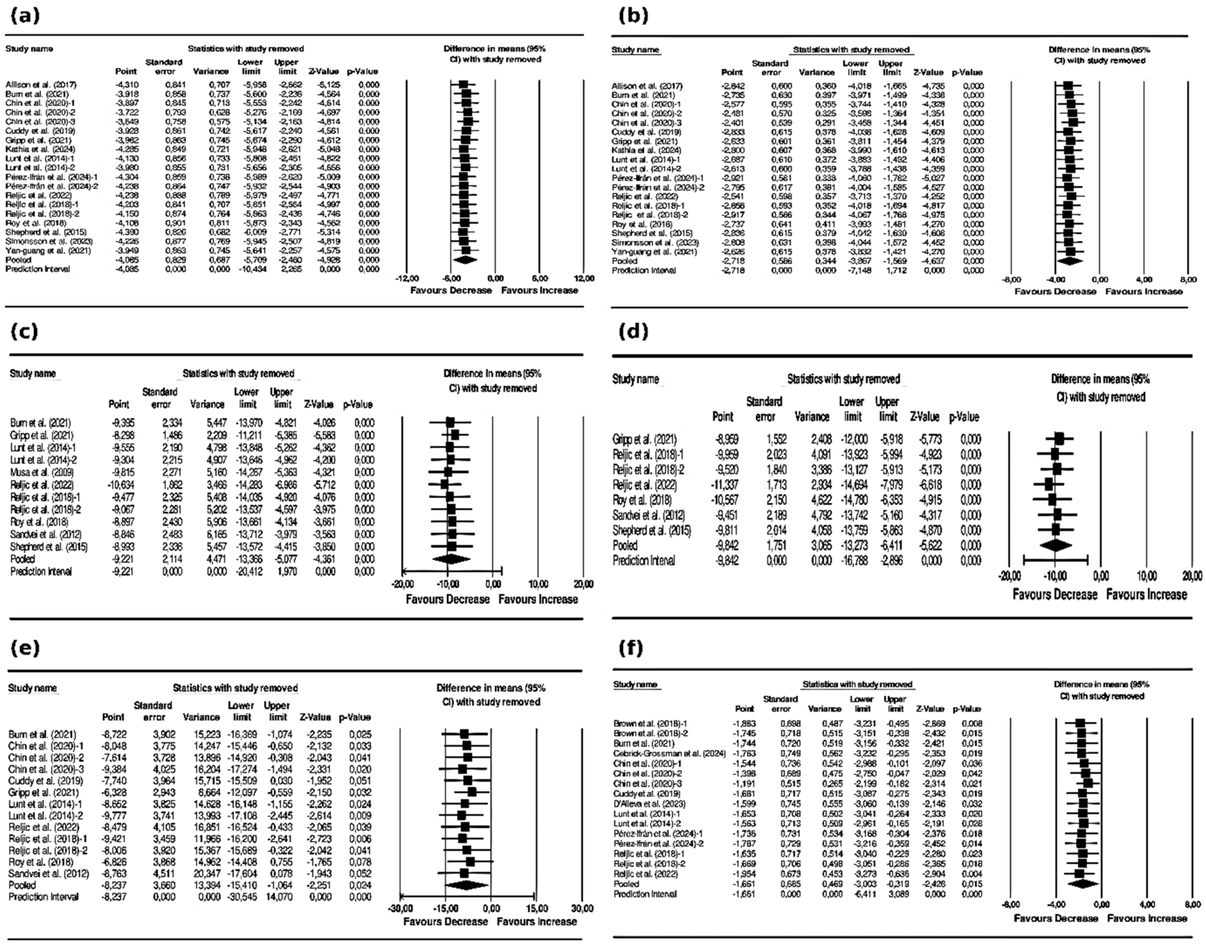

Figure S4. Forest plots of sensitivity analysis for the effects of real-world HIIT on (**a**) systolic blood pressure, (**b**) diastolic blood pressure, (**c**) total cholesterol, (**d**) LDL cholesterol, (**e**) triglycerides, and (**f**) waist circumference. Weighted mean differences and 95% confidence intervals.

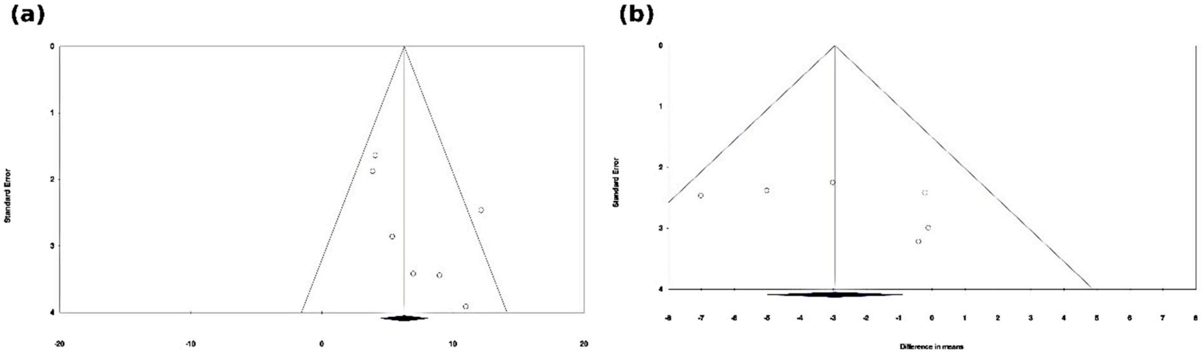

Figure S5. Funnel plots for the effects of real-world HIIT versus passive controls on (**a**) VO_{2max}, and (**b**) waist circumference.

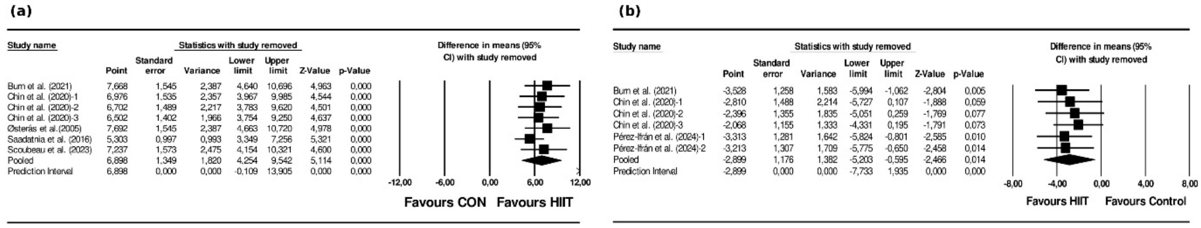

Figure S6. Forest plots of sensitivity analysis for the effects of real-world HIIT versus passive controls on (**a**) VO_{2max}, and (**b**) waist circumference. Weighted mean differences and 95% confidence intervals.

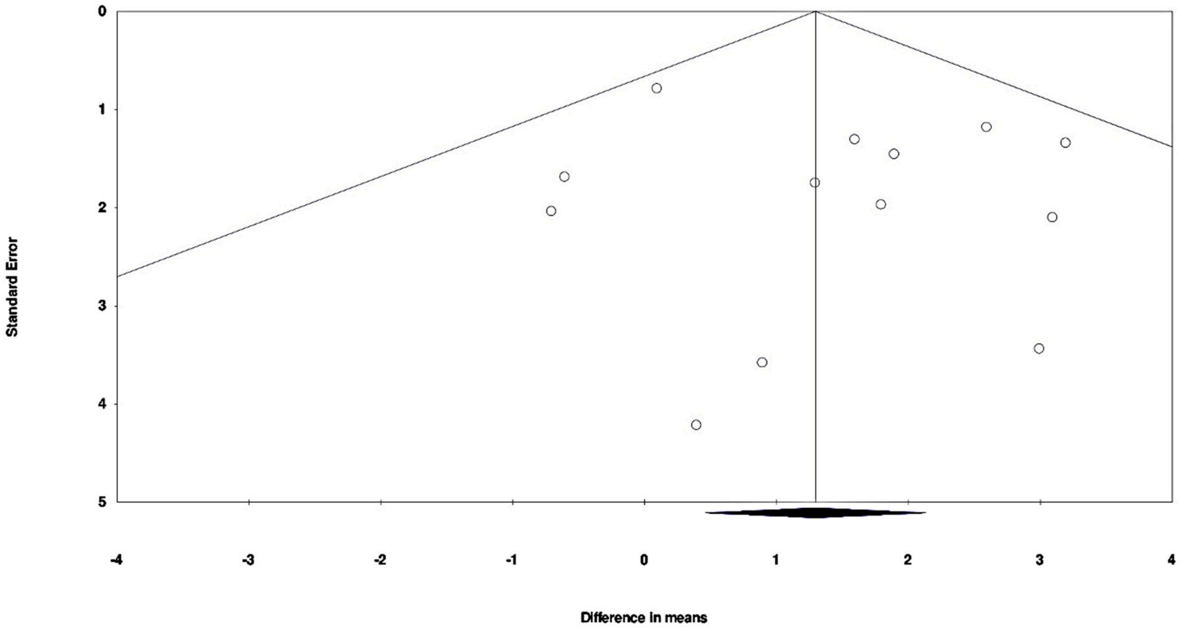

Figure S7. Funnel plot for the effects of real-world HIIT versus active controls on VO_{2max}.

Study name	Statistics with study removed						
	Point	Standard error	Variance	Lower limit	Upper limit	Z-Value	p-Value
Arboleda-Serna et al. (2022)	1,188	0,348	0,121	0,506	1,870	3,413	0,001
Cuddy et al. (2019)	1,109	0,348	0,121	0,427	1,790	3,190	0,001
Gripp et al. (2021)	0,974	0,352	0,124	0,283	1,665	2,764	0,006
Kathia et al. (2024)	1,095	0,346	0,120	0,417	1,774	3,163	0,002
Knappett et al. (2023)	1,168	0,346	0,120	0,490	1,846	3,377	0,001
Lunt et al. (2014)-1	1,063	0,346	0,119	0,386	1,740	3,076	0,002
Lunt et al. (2014)-2	1,071	0,351	0,123	0,384	1,758	3,055	0,002
Reljic et al. (2018)-1	1,097	0,343	0,117	0,426	1,769	3,202	0,001
Reljic et al. (2018)-2	1,118	0,343	0,117	0,447	1,789	3,264	0,001
Roy et al. (2018)	1,345	0,377	0,142	0,605	2,085	3,563	0,000
Sandvei et al. (2012)	1,295	0,427	0,182	0,458	2,131	3,034	0,002
Shepherd et al. (2015)	1,121	0,342	0,117	0,450	1,791	3,276	0,001
Song et al. (2024)-1	1,081	0,353	0,125	0,389	1,773	3,062	0,002
Song et al. (2024)-2	0,983	0,356	0,127	0,285	1,681	2,761	0,006
Pooled	1,116	0,341	0,116	0,448	1,784	3,273	0,001
Prediction Interval	0,000	0,000	0,000	0,000	0,000	0,000	0,000

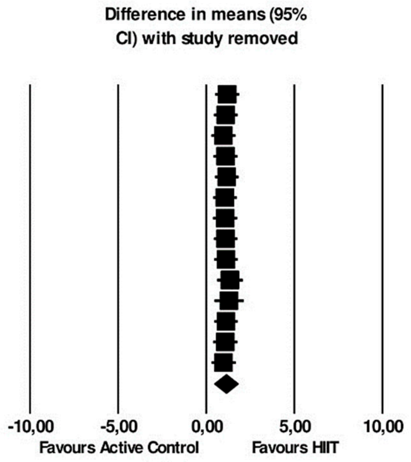

Figure S8. Forest plot of sensitivity analysis for the effects of real-world HIIT versus active controls on VO_{2max}. Weighted mean differences and 95% confidence intervals.
